# Lactylation shields S100A11 from degradation to promote gastric cancer metastasis

**DOI:** 10.1007/s13402-026-01255-w

**Published:** 2026-07-09

**Authors:** Qingrui Zhang, Yuhang Zhou, Fangning Wang, Jingrui Yan, Jiayu Liu, Lin Zhang, Shiyu Du

**Affiliations:** 1 Department of Gastroenterology, China-Japan Friendship Hospital, No. 2, Yinghua East Road, Chaoyang District, Beijing, 100029 China; 2 China-Japan Friendship Hospital (Institute of Clinical Medical Sciences), Chinese Academy of Medical Sciences & Peking Union Medical College, Beijing, 100029 China; 3 Department of Pathology, Beijing Huairou Hospital, Beijing, 101400 China; 4 Department of General Surgery, China-Japan Friendship Hospital, Beijing, 100029 China

**Keywords:** Gastric cancer, Lactylation, S100A11, FBXW11, Ubiquitination, Protein stability, Metastasis

## Abstract

**Background:**

Protein lactylation, a novel post-translational modification utilizing lactate as the substrate, has emerged as a key mechanism linking metabolic alterations to cancer progression. This study aims to investigate the role and underlying mechanism of S100A11 lactylation in gastric cancer progression and metastasis.

**Methods:**

Lactylome profiling, site-directed mutagenesis, co-IP, cycloheximide chase, ubiquitination assays, and in vitro/in vivo functional experiments were performed to dissect the regulatory effect of S100A11 lactylation.

**Results:**

S100A11 was identified as a hyperlactylated protein in gastric cancer tissues, with lysine 27 (K27) and lysine 55 (K55) serving as the primary modification sites. Mechanistically, lactylation at K27 and K55 disrupted the interaction between S100A11 and its E3 ubiquitin ligase FBXW11, thereby inhibiting polyubiquitination and proteasomal degradation of the protein. The K27R/K55R double mutation, which abolished both lactylation and ubiquitination at these residues, resulted in constitutive stabilization of S100A11. The stabilized S100A11 hyperactivated the PI3K-AKT and ERK1/2 signaling cascades and upregulated the downstream oncogenic effectors Cyclin D1 and c-Myc. Functionally, S100A11 stabilization significantly enhanced the proliferation, colony formation, migration and invasion of gastric cancer cells in vitro, and markedly promoted lung metastasis in vivo. Clinically, higher lactylation levels of S100A11 were observed in patients with advanced TNM stages.

**Conclusion:**

This study reveals a lactylation-ubiquitination competitive regulatory mechanism that governs S100A11 protein stability, which directly links Warburg effect-derived lactate to oncogenic protein stabilization and metastatic progression in gastric cancer.

**Graphical Abstract:**

**Figure:**
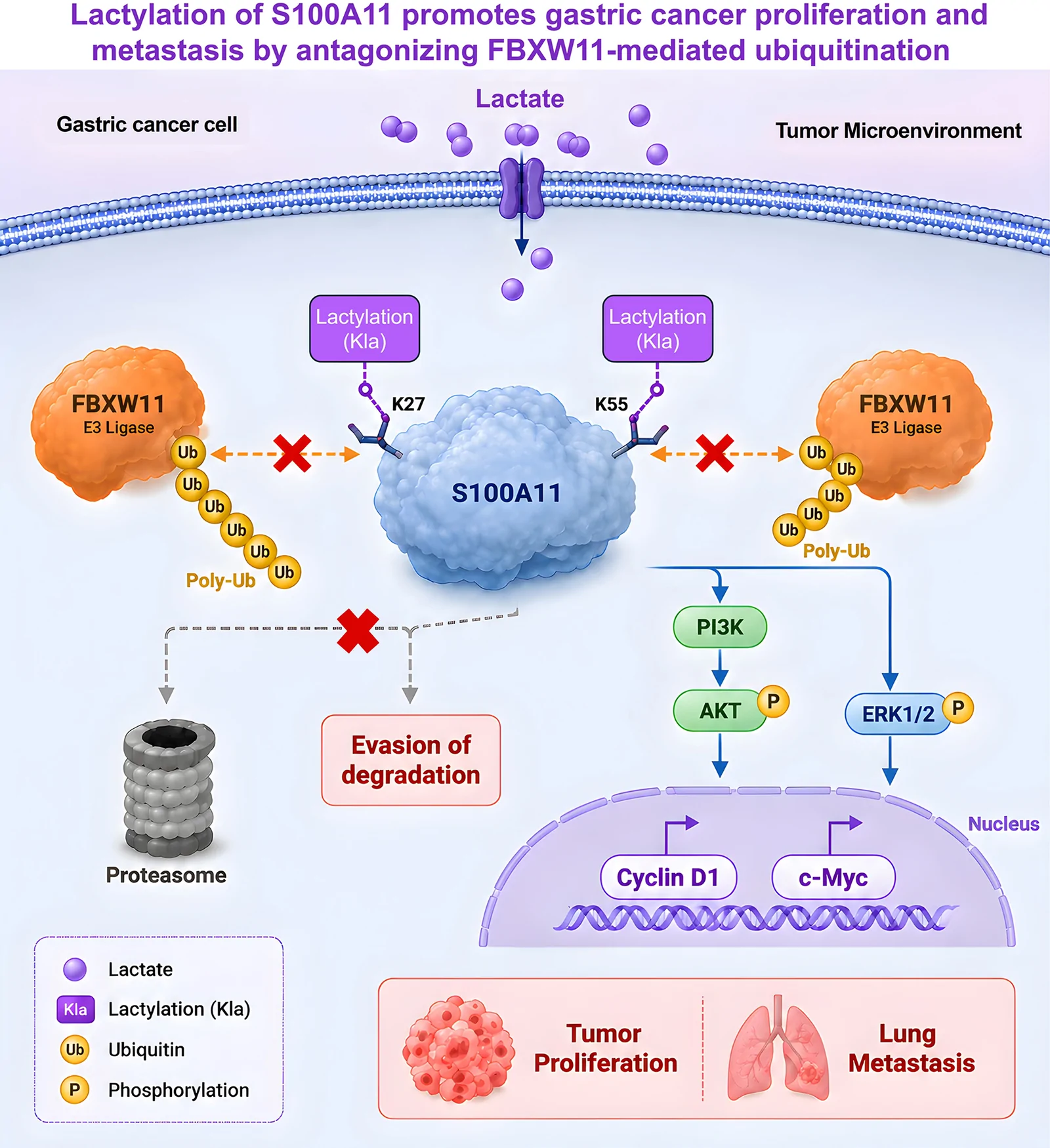
Tumor microenvironmental lactate induces lactylation of S100A11 at K27 and K55 in gastric cancer cells. The modification competitively abrogates FBXW11-mediated polyubiquitination at the same residues, thus stabilizing S100A11 protein. Accumulated S100A11 hyperactivates PI3K-AKT and ERK1/2 pathways to drive gastric cancer progression and lung metastasis.

**Supplementary Information:**

The online version contains supplementary material available at https://doi.org/10.1007/s13402-026-01255-w.

## Introduction

Gastric cancer (GC) is a highly aggressive malignancy and a leading cause of cancer-related mortality worldwide [1], largely due to its propensity for rapid metastasis and therapeutic resistance [2]. A defining hallmark of the GC tumor microenvironment (TME) is profound metabolic reprogramming, particularly the Warburg effect, which results in the massive accumulation of lactate [2]. Lactate is now recognized as a critical signaling molecule that actively shapes tumor progression [3]. Recently, protein lactylation-a novel post-translational modification (PTM) utilizing lactate as a substrate-has emerged as a key mechanism linking altered metabolism to cancer phenotype [4, 5]. While histone lactylation is known to regulate gene transcription, the scope and functional consequences of non-histone protein lactylation in driving GC metastasis remain poorly understood.

The S100 family of calcium-binding proteins is deeply implicated in gastrointestinal cancer progression, with distinct members exerting multifaceted roles in tumor invasion, immune evasion, and anoikis resistance [6]. Among these, S100A11 is frequently overexpressed in GC and consistently correlates with poor overall and progression-free survival, serving as a robust independent prognostic biomarker [7, 8]. Extensive research indicates that S100A11 functions as a potent oncogenic driver, promoting epithelial-mesenchymal transition (EMT), chemotherapy resistance, and invasion through the activation of NF-κB, matrix metalloproteinases (MMPs), and various growth factor pathways [7, 9]. However, while the transcriptional regulation and downstream effectors of S100A11 are well-documented, the synergistic relationship between its transcriptional upregulation and post-translational stabilization, as well as how this stability is dictated by the lactate-rich TME, remain unclear.

Intracellular S100A11 levels are tightly controlled by the ubiquitin-proteasome system, specifically through recognition by the E3 ubiquitin ligase FBXW11 [10], which mediates its degradation. Because lysine residues are frequent targets for both ubiquitination and novel acylations like lactylation, PTM crosstalk at these sites can dictate protein fate [11, 12]. We hypothesized that the metabolic shift in GC directly disrupts S100A11 turnover, wherein lactate-induced lactylation competitively inhibits its ubiquitination, thereby stabilizing the protein and sustaining oncogenic signaling.

Here, utilizing integrated lactylome profiling of primary GC tissues, we identify S100A11 as a heavily lactylated protein in the tumor compartment. We map the primary lactylation-associated lysines to K27 and K55 and provide evidence these specific residues serve as the direct dual-targets for both lactylation and FBXW11-mediated polyubiquitination. Consequently, lactylation at these sites imposes spatial steric hindrance that interferes with FBXW11-mediated recognition and subsequent ubiquitination of S100A11. This evasion of proteasomal degradation leads to the stabilization of S100A11, which in turn hyperactivates the PI3K-AKT and ERK1/2 signaling cascades. Consequently, the constitutively stabilized state of S100A11 (which phenocopies the lactylated state by evading ubiquitin-mediated degradation) drives aggressive proliferation, invasion, and metastatic dissemination in vitro and in vivo. Therefore, our study elucidates a mechanistic axis linking the Warburg effect to protein stability and downstream oncogenic signaling, highlighting S100A11 lactylation as a critical vulnerability and a promising therapeutic target in metastatic gastric cancer.

## Materials and methods

A detailed description of all methods is provided in the Supplementary Materials. Key procedures are summarized below.

### Bioinformatic analysis

The study utilized integrated lactylome data from a published cohort of primary gastric cancer (GC) and matched normal tissues (*n* = 10 per group) [13]. Gene expression and survival analyses for S100 family members were performed using TCGA-STAD RNA-seq data [14] via the UCSC Xena Browser [15] and the Kaplan-Meier plotter [16], respectively. Protein structure was predicted using AlphaFold 3 (https://alphafoldserver.com/) [17].

### Ethics and tissue samples

Primary GC tissues for validation were collected from patients (*n* = 6) with informed consent under IRB approval (2023-KY-174).

### Cell culture and reagents

Human GC cell lines AGS and MKN-45 were cultured in RPMI-1640 with 10% FBS. Cells were treated with sodium L-lactate (0–20 mM), SAHA (5 µM), or CTB (10 µM) as indicated.

### Plasmid construction and stable cell line generation

Wild-type (WT) and mutant (K27R, K55R, K27R/K55R) *S100A11* cDNA sequences were cloned into a Flag-tagged lentiviral vector. Knockdown was achieved using shRNA targeting *S100A11* (shS100A11) or a non-targeting control (shNC). Stable knockdown and rescue cell lines were generated via sequential lentiviral transduction and antibiotic selection.

### Protein analysis

Protein lactylation and expression were analyzed by co-immunoprecipitation (Co-IP) and western blotting using specific antibodies (listed in Supplementary Materials). The protein stability of S100A11 was assessed by cycloheximide (CHX) chase assay over a 36-h time course. Endogenous ubiquitination was strictly evaluated by IP-western blot under stringent denaturing conditions (1% SDS and boiling) after MG132 treatment to rule out non-covalent interactions. Protein-protein interactions, particularly the highly dynamic and transient E3 ligase-substrate complex (FBXW11-S100A11), were examined by Co-IP under optimized mild lysis conditions to preserve the integrity of the protein assemblies. A comprehensive description of these procedures and controls is available in the Supplementary Methods.

### Functional assays

Cell proliferation was measured by CCK-8 assay. Colony formation, migration, and invasion were assessed using crystal violet staining and Transwell chambers.

### In Vivo metastasis model

Animal studies were approved by the ethics committee of China-Japan Friendship Hospital, China (ZRDWLL260017). A tail vein injection model was established using BALB/c nude mice. Mice were injected with AGS cells (shNC, shS100A11, or shS100A11 cells rescued with WT or K27R/K55R S100A11). Lung metastatic burden was quantified from H&E-stained sections after 21 days.

### Statistical analysis

Data are presented as mean ± SD. Comparisons between two groups used Student’s t-test; comparisons among multiple groups used one-way ANOVA with Tukey’s post-hoc test. Survival analysis used the log-rank test. *P* < 0.05 was considered significant.

## Results

### Identification of differentially lactylated S100 proteins in gastric cancer

**Fig. 1:**
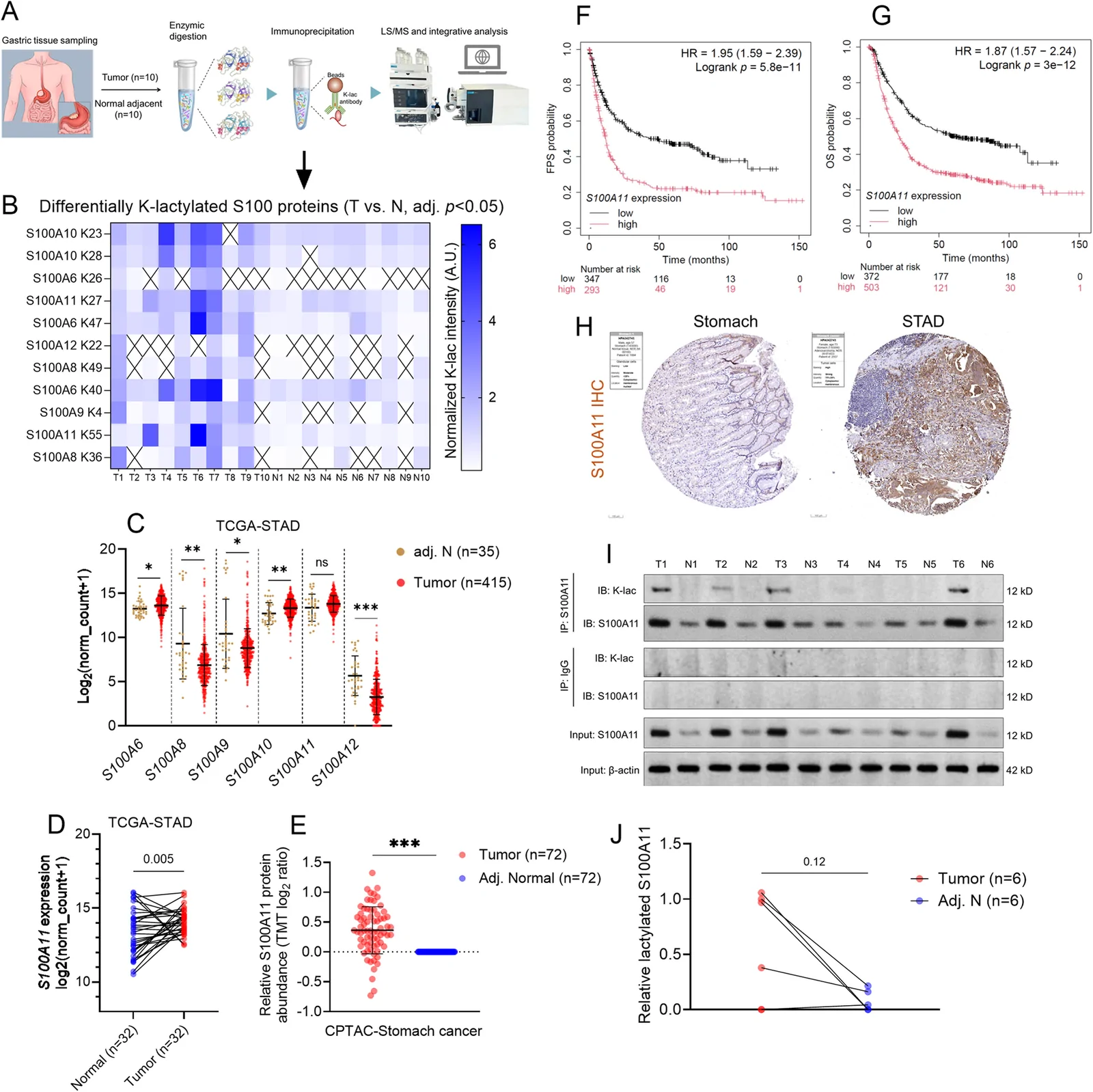
Identification and validation of differentially lactylated S100 proteins in gastric cancer. **(A)** Schematic workflow for lactylome profiling in gastric cancer and adjacent normal tissues, including enzymatic digestion, immunoprecipitation, and LC-MS/MS analysis. **(B)** Heatmap showing normalized K-lac intensity of S100 proteins with significant differential lactylation (adj. *p* < 0.05) between tumor (T) and normal (N) tissues. **(C)** Comparison of S100 gene expression levels in gastric tumor (*n* = 415) and adjacent normal tissues (*n* = 35) from TCGA-STAD RNA-seq data. **(D)** Paired analysis of *S100A11* mRNA expression in matched tumor and normal tissues (*n* = 32) from the TCGA-STAD dataset. **(E)** Comparison of S100A11 protein expression levels in gastric tumor and matched normal tissues (*n* = 72) from the CPTAC database. **(F**,** G)** Kaplan-Meier survival curves for first progression-free survival (FPS, F) and overall survival (OS, G) based on *S100A11* expression in gastric cancer patients. **(H)** Immunohistochemical staining of S100A11 in normal stomach and gastric cancer (STAD) tissues from the Human Protein Atlas. Images were accessed with credit from the HPA, via: https://images.proteinatlas.org/42745/106098_A_5_1.jpg and https://images.proteinatlas.org/42745/106096_B_1_5.jpg. **(I)** Co-IP assays to detect S100A11 lactylation in primary gastric cancer tissues (*n* = 6). **(J)** Quantitative analysis of relative S100A11 lactylation levels (K-lac intensity normalized to total immunoprecipitated S100A11) from the Co-IP assays (*n* = 5 pairs; one was excluded from quantification due to no signal in both groups). Data were presented as mean ± SD. *p < 0.05, **p < 0.01, ***p < 0.001

Using integrated lactylome profiling based on a recent dataset covering gastrointestinal cancers (Fig. 1A), we identified several S100 family proteins with significantly elevated lactylation in gastric tumor tissues compared to normal tissues, including S100A6, S100A8, S100A9, S100A10, S100A11, and S100A12 (Fig. 1B). Then, we examined the expression profiles of the genes encoding these proteins, based on RNA-seq data from gastric cancer and adjacent normal tissues in TCGA-STAD (Fig. 1C). *S100A6* and *S100A10* were significantly upregulated, while *S100A8*,* S100A9*, and *S100A12* were significantly downregulated in the tumor group (Fig. 1C). *S100A11* showed similar expression between the two groups (Fig. 1C). However, given previous reports of elevated *S100A11* mRNA in gastric cancer [7, 18], we further performed a paired analysis of matched tumor and adjacent normal tissues within the TCGA-STAD dataset (*n* = 32). This analysis revealed a significant upregulation of *S100A11* mRNA in tumor tissues (Fig. 1D, *p* = 0.005), which is consistent with the literature [7, 18]. We further analyzed its protein expression using the CPTAC-STAD dataset. Notably, S100A11 protein levels were significantly higher in gastric tumor tissues than in matched normal tissues (Fig. 1E, *p* < 0.001). The robust elevation of S100A11 protein abundance, considered alongside our lactylation data, suggests that while transcriptional upregulation contributes to its expression, post-translational modifications (such as hyper-lactylation) likely play a synergistic and crucial role in further stabilizing and accumulating S100A11 protein in gastric cancer.

Survival analysis using the Kaplan-Meier plotter demonstrated that among these genes (Supplementary Figure 1), only high *S100A11* expression is associated with reduced first progression-free survival (FPS, Fig. 1F) and overall survival (OS, Fig. 1G) in gastric cancer patients. Immunohistochemical staining confirmed elevated S100A11 protein levels in gastric cancer tissues compared to normal stomach (Fig. 1H). Co-IP assays further confirmed increased lactylation of S100A11 in a subset (*n* = 4) of primary gastric cancer cases (Fig. 1I). Subsequent quantitative analysis revealed a trend of increased lactylation of S100A11 in primary gastric cancer tissues compared to adjacent normal tissues (Fig. 1J). Although the statistical difference was marginal (*p* = 0.12) likely due to the limited sample size, the overall pattern supports the hyper-lactylation of S100A11 in some of the gastric cancer tissues.

### Lactate induces S100A11 lactylation in gastric cancer cells

**Fig. 2:**
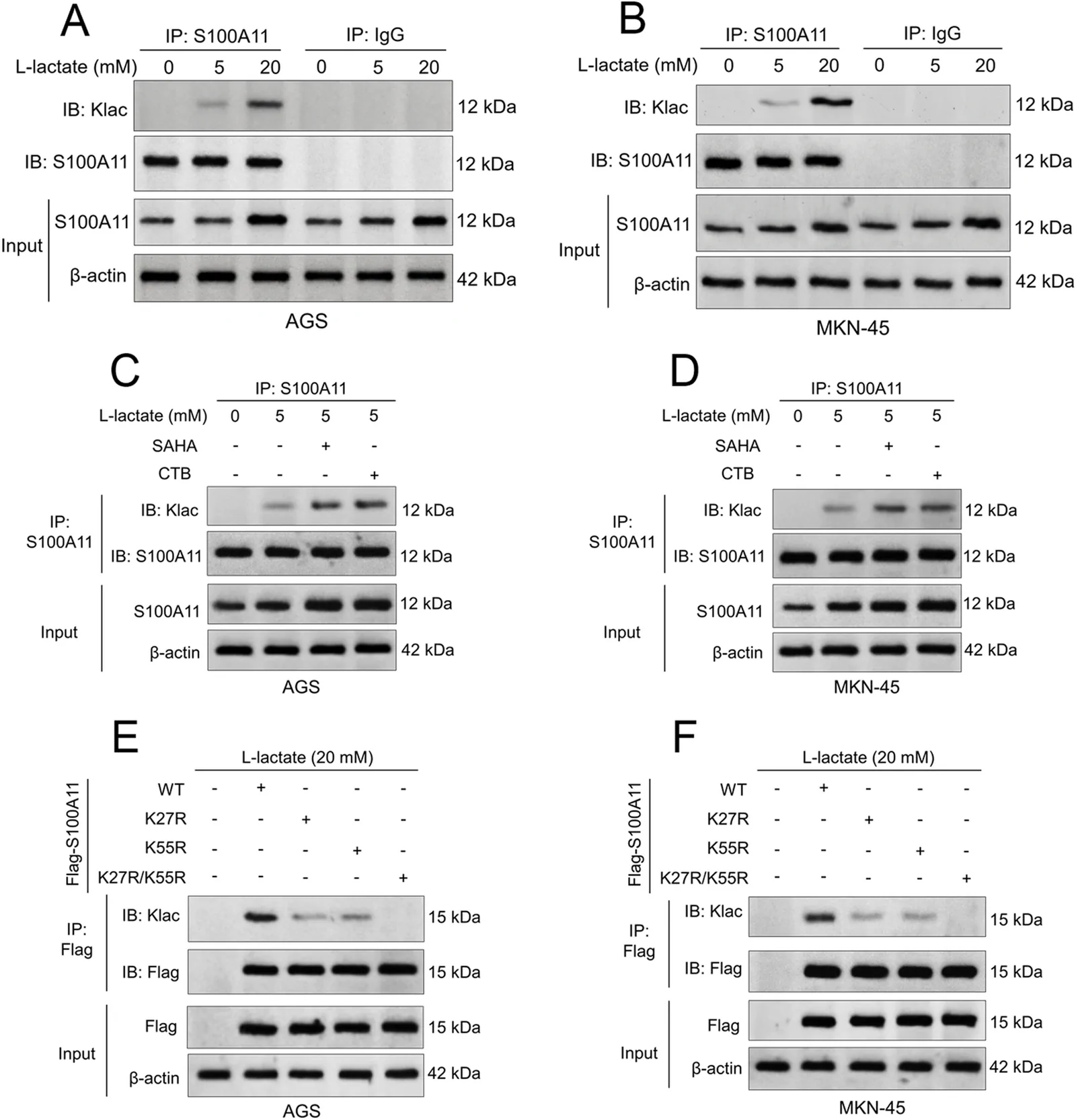
Validation and site identification of S100A11 lactylation in gastric cancer cells. **(A**,** B)** AGS **(A)** and MKN-45 **(B)** cells were treated with increasing concentrations of L-lactate (0, 5, 20 mM) for 24 h. Endogenous S100A11 was immunoprecipitated and analyzed by immunoblotting with anti-lactyllysine (K-lac) and anti-S100A11 antibodies. IgG was used as a negative control. **(C**,** D)** AGS **(C)** and MKN-45 **(D)** cells were treated with 5 mM L-lactate in the presence or absence of the HDAC inhibitor SAHA (5 µM) or p300 activator CTB (10 µM) for 24 h. S100A11 lactylation was assessed as above. **(E**,** F)** AGS **(E)** and MKN-45 **(F)** cells were stably transduced with Flag-tagged wild-type (WT) or lysine-to-arginine mutants (K27R, K55R, K27R/K55R) of S100A11, followed by treatment with or without 20 mM L-lactate for 24 h. Flag-S100A11 was immunoprecipitated (IP: Flag) and analyzed for lactylation. Input samples were probed for Flag and β-actin

To validate the lactylation of S100A11, AGS and MKN-45 gastric cancer cells were treated with increasing concentrations of L-lactate. Immunoprecipitation of endogenous S100A11 followed by immunoblotting with an anti-lactyllysine antibody revealed a dose-dependent increase in S100A11 lactylation in both cell lines (Fig. 2A-B). No signal was detected in the IgG controls.

To investigate the regulatory mechanisms of S100A11 lactylation, we aimed to determine whether this modification is an enzymatically driven, dynamic process. Recent studies have established that protein lactylation shares enzymatic regulators with acetylation, where p300 acts as a lactyltransferase (“writer”) and classical histone deacetylases (HDACs) function as delactylases (“erasers”) [5, 19]. Based on this rationale, AGS and MKN-45 cells were treated with the HDAC inhibitor SAHA or the p300 activator CTB in the presence of L-lactate. SAHA or CTB treatment further enhanced S100A11 lactylation (Fig. 2C-D).

To determine the specific lysine residues involved in S100A11 lactylation, Flag-tagged expression vectors containing chemically synthesized wild-type or mutant (K27R, K55R, and K27R/K55R) cDNAs were transfected into AGS and MKN-45 cells. Following L-lactate treatment, immunoprecipitation and immunoblotting showed that mutation of either K27 or K55 partially reduced S100A11 lactylation, while the double mutant (K27R/K55R) almost completely abolished lactylation, indicating that both K27 and K55 are major lactylation sites on S100A11 (Fig. 2E-F).

### S100A11-K27R/K55R reduces its interaction with FBXW11 and FBXW11-mediated proteasomal degradation in gastric cancer cells

**Fig. 3:**
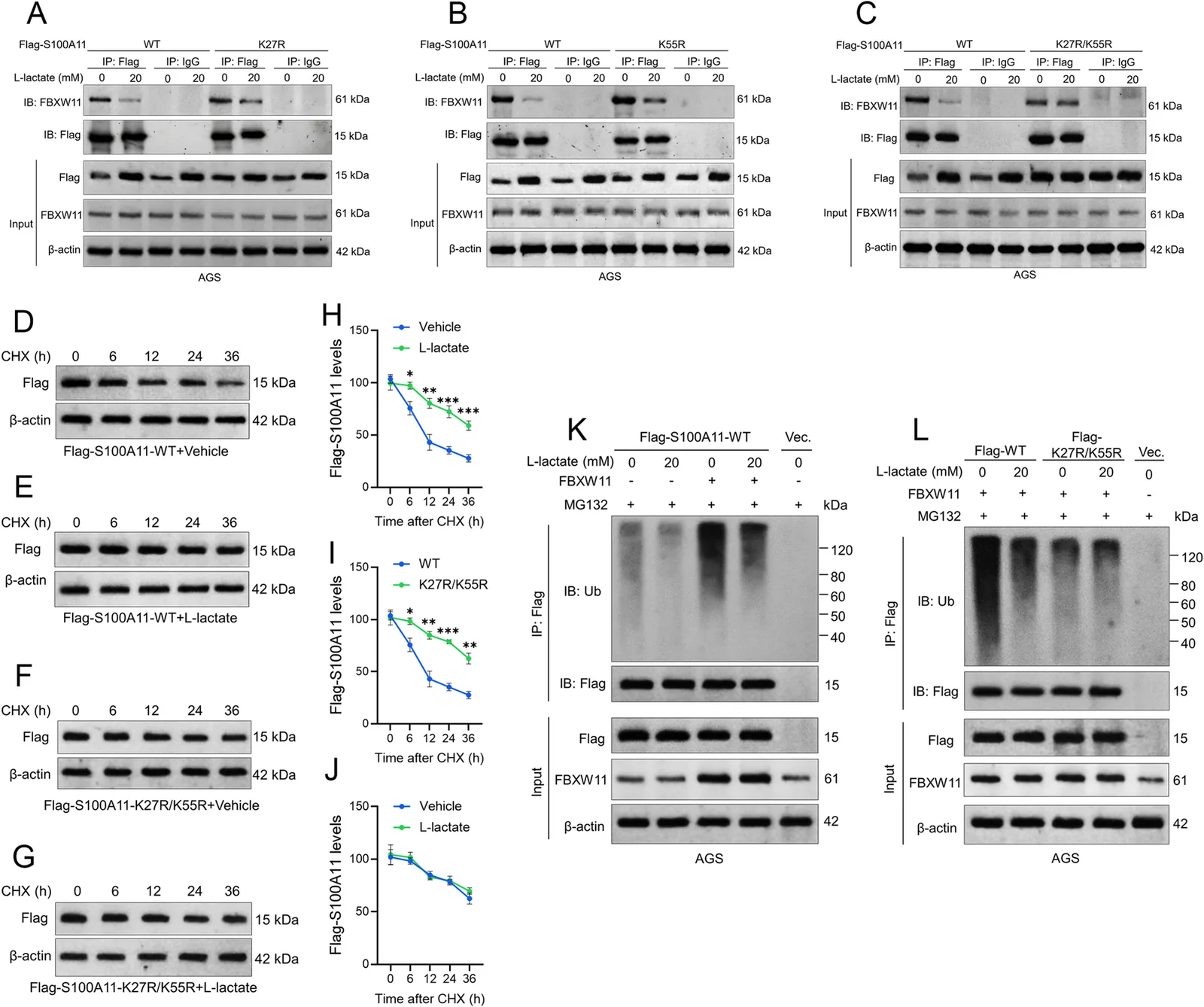
S100A11 lactylation at K27 and K55 reduces its interaction with FBXW11 and FBXW11-mediated proteasomal degradation in gastric cancer cells. **(A-C)** Co-immunoprecipitation of Flag-S100A11-WT, the single mutants K27R **(A)** and K55R **(B)**, or the K27R/K55R double mutant **(C)** with FBXW11 in AGS cells treated with or without 20 mM L-lactate for 24 h. Cell lysates were immunoprecipitated with anti-Flag magnetic beads and analyzed by immunoblotting with anti-FBXW11 and anti-Flag antibodies. Corresponding IgG groups with or without L-lactate treatment were included in parallel to exclude any non-specific protein binding. **(D-J)** Images **(D-G)** and quantitation **(H-J)** of Cycloheximide (CHX) chase assays in AGS cells expressing Flag-S100A11-WT or K27R/K55R treated with vehicle or 20 mM L-lactate. Cells were harvested at the indicated times after CHX addition, and Flag-S100A11 levels were analyzed by western blotting. **(K-L)** In vivo endogenous ubiquitination assays in AGS cells expressing Flag-S100A11-WT **(K)** or K27R/K55R **(L)**. Cells were treated with or without 20 mM L-lactate for 24 h, followed by treatment with 10 µM MG132 for 6 h before harvest to block proteasomal degradation. Cell lysates were subjected to denaturing immunoprecipitation with anti-Flag magnetic beads, and endogenous ubiquitinated S100A11 was detected by immunoblotting with an anti-ubiquitin antibody. Input samples were probed for FBXW11, Flag, and β-actin. Data are presented as mean ± SD; *p < 0.05, ** *p* < 0.01, * *p* < 0.001

S100A11 can be degraded by E3 ubiquitin ligase FBXW11-mediated proteasomal degradation [10]. To investigate whether S100A11 lactylation affects its interaction with FBXW11, we performed co-immunoprecipitation assays in AGS gastric cancer cells expressing Flag-tagged S100A11-WT, the individual single mutants (K27R or K55R), or the K27R/K55R double mutant. Lactate treatment markedly reduced the interaction between S100A11-WT and FBXW11. Importantly, both the K27R and K55R single mutants maintained a strong interaction with FBXW11 under basal conditions, which was only partially diminished by lactate treatment, indicating that lactylation at a single site is insufficient to fully block FBXW11 binding (Fig. 3A-B). Furthermore, the K27R/K55R mutant, which is refractory to both lactylation and ubiquitination, exhibited a constitutive, strong binding to FBXW11 regardless of lactate exposure (Fig. 3C). To exclude the possibility that this altered interaction was an artifact of protein misfolding, we performed structural simulations using AlphaFold 3 (Supplementary Figure 2). The modeling confirmed that the K27R/K55R substitution preserves the overall 3D structural scaffold of S100A11 identical to that of the wild-type. These results suggest that lactylation at both K27 and K55 provides spatial steric hindrance that blocks FBXW11 recognition.

To assess the impact of S100A11 lactylation on protein stability, cycloheximide (CHX) chase assays were performed. In cells expressing S100A11-WT, lactate treatment significantly delayed protein degradation compared to the vehicle control (Fig. 3D, E, H). Interestingly, despite exhibiting a strong, constitutive interaction with FBXW11 (Fig. 3C), the K27R/K55R mutant paradoxically displayed markedly increased stability compared to S100A11-WT, and its degradation rate remained unaffected by lactate treatment (Fig. 3F, G, I, J). Moreover, CHX assays of the individual single mutants revealed an intermediate phenotype: mutating a single site (retaining one intact lysine) resulted in partial stabilization under basal conditions, and their degradation could only be partially rescued by lactate treatment (Supplementary Figure 3). This striking uncoupling of E3 ligase binding from protein degradation in the K27R/K55R mutant led us to hypothesize that K27 and K55 might serve a dual role: acting not only as sites for lactylation but also as the direct lysine acceptor sites for FBXW11-mediated polyubiquitination.

To directly test this hypothesis, in vivo ubiquitination assays in the presence of the proteasome inhibitor MG132 were performed. The results demonstrated that lactate treatment substantially reduced the endogenous polyubiquitination of S100A11-WT (Fig. 3K). In contrast, despite its constant association with FBXW11, the K27R/K55R mutant maintained a dramatically reduced basal level of ubiquitination that was unresponsive to lactate treatment (Fig. 3L). These findings confirm that K27 and K55 are the target residues for ubiquitination, and their mutation to arginine abolishes ubiquitin conjugation. Lactylation at K27 and K55 competitively blocks FBXW11 recognition and subsequent ubiquitination at the same residues, thereby providing a physiological mechanism for S100A11 stabilization in the lactate-rich tumor microenvironment.

### S100A11-K27R/K55R mutant enhances the activation of PI3K-AKT and ERK1/2 signaling pathways in gastric cancer cells

**Fig. 4:**
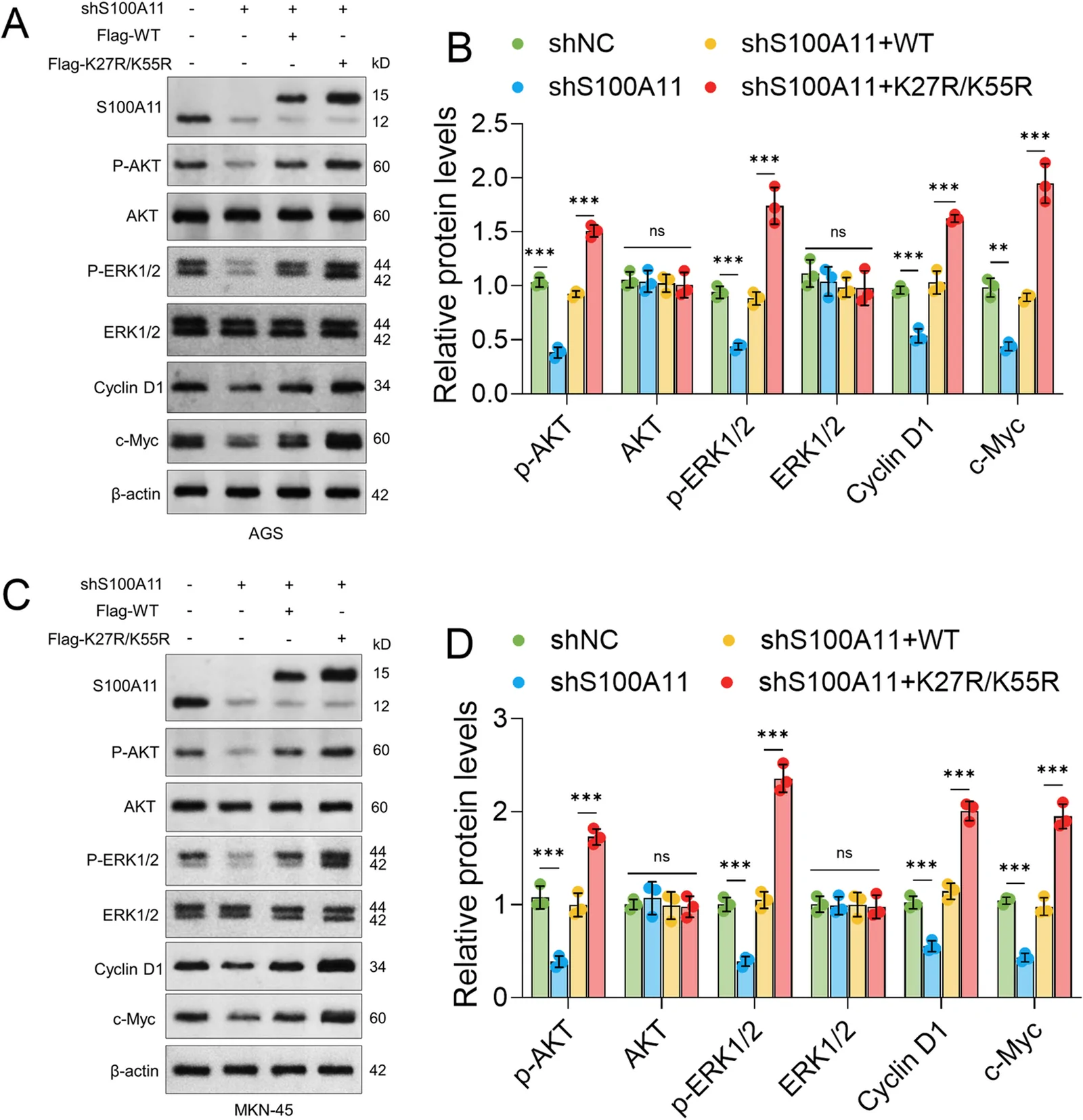
The S100A11-K27R/K55R mutant enhances activation of PI3K-AKT and ERK1/2 signaling pathways in gastric cancer cells. **(A**,** C)** Western blot analysis of S100A11, phosphorylated AKT (p-AKT), total AKT, phosphorylated ERK1/2 (p-ERK1/2), total ERK1/2, Cyclin D1, c-Myc, and β-actin in AGS **(A)** and MKN-45 **(C)** cells. Cells were transduced with control shRNA (shNC), S100A11 shRNA (shS100A11), or S100A11 shRNA followed by rescue with Flag-tagged wild-type S100A11 (shS100A11 + WT) or K27R/K55R mutant (shS100A11 + K27R/K55R). **(B**,** D)** Quantification of relative protein levels of p-AKT, AKT, p-ERK1/2, ERK1/2, Cyclin D1, and c-Myc in AGS **(B)** and MKN-45 **(D)** cells, normalized to β-actin. Data are presented as mean ± SD, *n* = 3; *p < 0.05, **p < 0.01, ***p < 0.001

Previous studies have established that S100A11 is closely linked to the activation of key oncogenic cascades, including AKT phosphorylation [20, 21] and prolonged MAP kinase (ERK1/2) signaling [20, 22]. Given our finding that the K27R/K55R mutant exhibits constitutive protein stabilization by evading FBXW11-mediated degradation, we hypothesized that this highly stable form would exert a more profound effect on these specific downstream networks. AGS and MKN-45 cells were first depleted of endogenous S100A11 using shRNA, and then rescued with either Flag-tagged wild-type S100A11 (WT) or the K27R/K55R double mutant. Knockdown of S100A11 significantly reduced the levels of phosphorylated AKT (p-AKT), phosphorylated ERK1/2 (p-ERK1/2), Cyclin D1, and c-Myc compared to control cells (Fig. 4A-D). Re-expression of S100A11-WT effectively restored the activation of these pathways, whereas expression of the K27R/K55R mutant resulted in a much stronger increase in p-AKT, p-ERK1/2, Cyclin D1, and c-Myc levels compared to WT rescue (Fig. 4A-D). Total AKT and ERK1/2 protein levels remained unchanged across all groups. Densitometric quantification of the bands confirmed that the K27R/K55R mutant had a significantly greater effect on the activation of PI3K-AKT and ERK1/2 signaling than wild-type S100A11 in both AGS and MKN-45 cells (Fig. 4B, D). These results suggest that the constitutive stabilization of S100A11 via the K27R/K55R mutation (which uncouples it from ubiquitin-mediated degradation) enhances its ability to activate oncogenic signaling pathways in gastric cancer cells.

### S100A11-K27R/K55R mutant enhances the proliferation, colony formation, migration, and invasion of gastric cancer cells

**Fig. 5:**
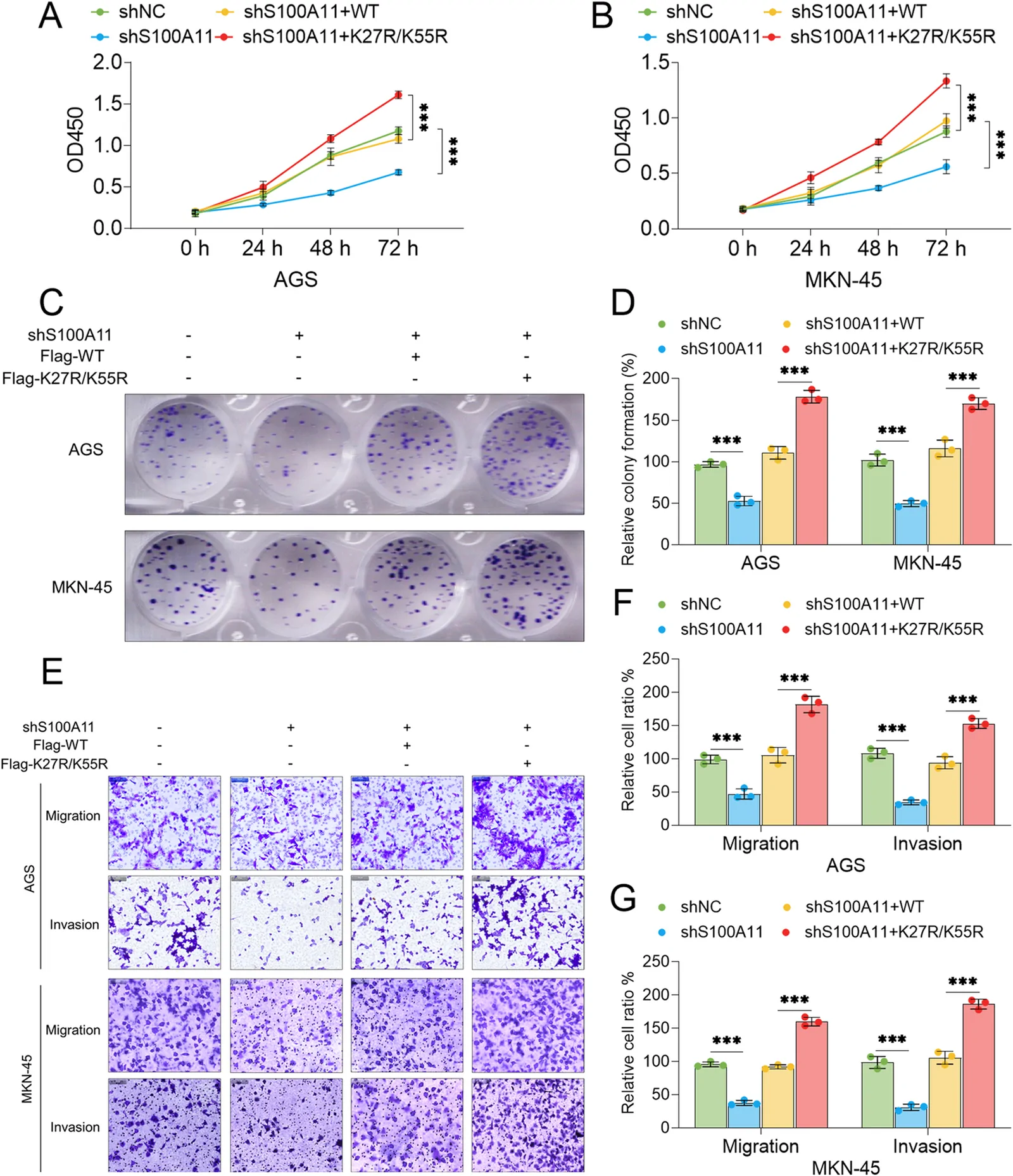
The S100A11-K27R/K55R mutant enhances the proliferation and invasion of gastric cancer cells. **(A**,** B)** CCK-8 assays showing cell proliferation of AGS **(A)** and MKN-45 **(B)** cells with S100A11 knockdown (shS100A11) and rescue by wild-type (WT) or K27R/K55R mutant S100A11. **(C**,** D)** Representative images **(C)** and quantification **(D)** of colony formation assays in AGS and MKN-45 cells with the indicated treatments. **(E-G)** Representative images **(E)** and quantification **(F, G)** of transwell migration and invasion assays in AGS **(F)** and MKN-45 **(G)** cells. Data are presented as mean ± SD. *p < 0.05, **p < 0.01, ***p < 0.001

Next, we investigated the functional consequences of the constitutively stabilized S100A11 mutant on gastric cancer cell behavior. *S100A11* knockdown significantly suppressed cell proliferation, while re-expression of wild-type S100A11 effectively restored proliferation rates. Notably, the K27R/K55R mutant led to a much greater increase in proliferation compared to wild-type rescue in both AGS and MKN-45 cells (Fig. 5A-B).

Consistently, colony formation assays showed that *S100A11* knockdown markedly reduced the number of colonies formed, which was partially rescued by wild-type S100A11 and further enhanced by the K27R/K55R mutant (Fig. 5C-D).

Transwell migration and invasion assays demonstrated that *S100A11* knockdown significantly impaired both migratory and invasive capacities of gastric cancer cells. While wild-type S100A11 re-expression effectively restored these abilities, the K27R/K55R mutant resulted in a significantly higher increase in migration and invasion compared to wild-type rescue (Fig. 5E-G).

### S100A11-K27R/K55R mutant enhances the metastasis of AGS cells in vivo

**Fig. 6:**
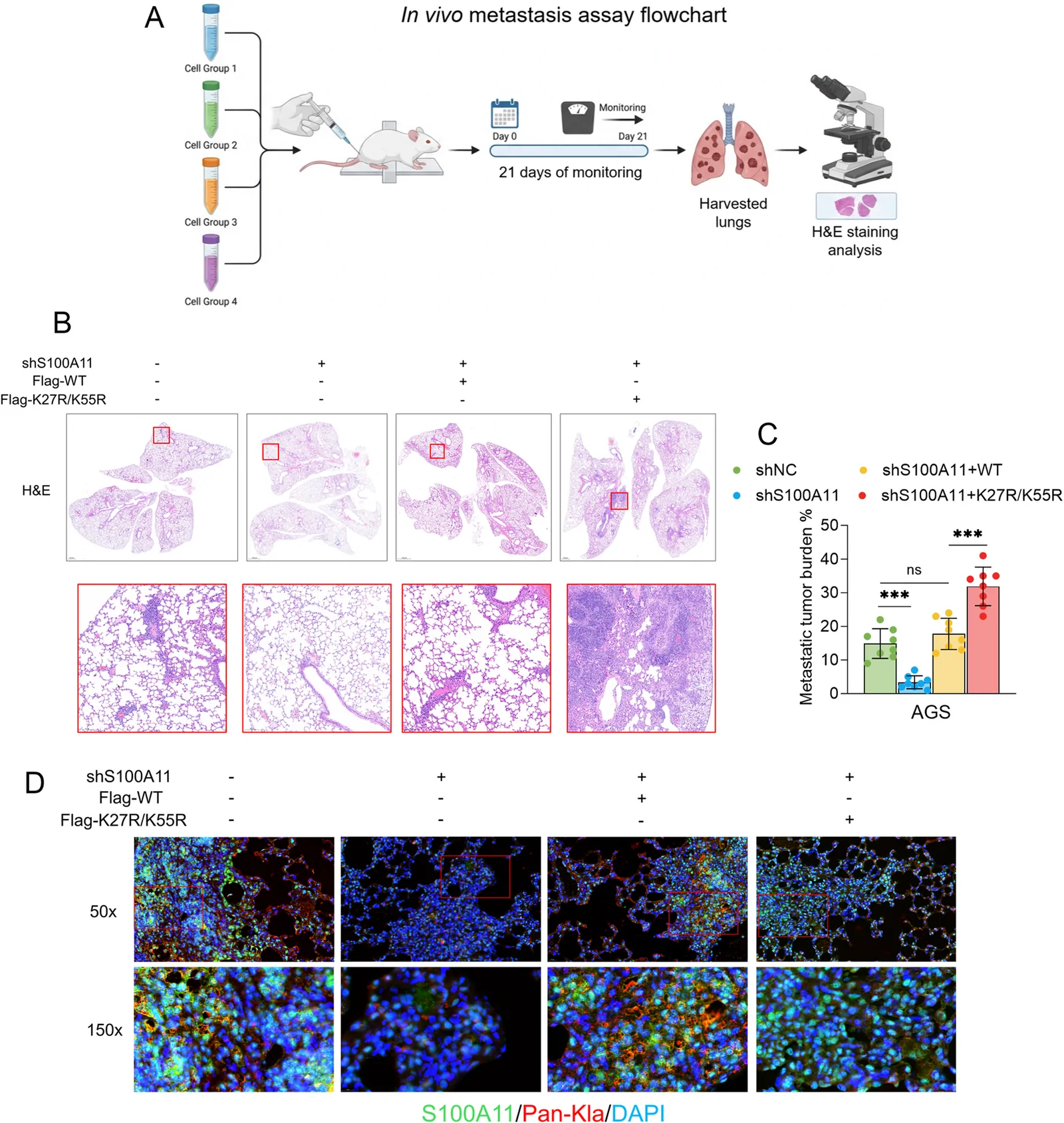
The S100A11-K27R/K55R mutant enhances the metastasis of AGS cells in vivo. **(A)** Schematic diagram of the in vivo metastasis assay. AGS cells with S100A11 knockdown and rescue by wild-type (WT) or K27R/K55R mutant S100A11 were injected into the tail vein of nude mice. After 21 days, lungs were harvested for H&E staining. **(B)** Representative H&E-stained lung sections showing metastatic nodules (upper: low magnification; lower: high magnification of boxed areas). **(C)** Quantification of metastatic tumor burden (*n* = 8) in the lungs (percentage of metastatic area per lung section). **(D)** Representative multiplex immunofluorescence (mIHC) images of lung metastatic nodules from the indicated groups. Tissue sections were co-stained for S100A11 (green), Pan-Kla (red), and nuclei (DAPI, blue). The merged yellow/orange signals indicate the in vivo co-localization of S100A11 and pan-lactylation. The boxed areas in the 50x images are shown at higher magnification (150x) in the lower panels. Data are presented as mean ± SD. *p < 0.05, **p < 0.01, ***p < 0.001

To evaluate the effect of the constitutively stabilized S100A11 on gastric cancer metastasis in vivo, we established a tail vein injection metastasis model using AGS cells. Mice were injected with the indicated cells and monitored for 21 days before lung tissues were harvested for histological analysis (Fig. 6A), H&E staining revealed that *S100A11* knockdown significantly reduced the number and size of metastatic nodules in the lungs compared to the control group. Re-expression of wild-type S100A11 effectively restored lung metastasis, while the K27R/K55R mutant led to a further increase in metastatic tumor burden (Fig. 6B). Quantitative analysis confirmed that the metastatic tumor burden was significantly higher in the K27R/K55R group compared to the wild-type rescue group (Fig. 6C).

To directly validate the in vivo occurrence of S100A11 lactylation within the tumor microenvironment, we performed multiplex immunofluorescence (mIHC) co-staining on the harvested lung metastatic tissues. As shown in Fig. 6D, both S100A11 and pan-lactyllysine (pan-Kla) signals were highly enriched within the dense metastatic tumor nodules compared to the surrounding normal alveolar structures. Crucially, in the tumors formed by wild-type S100A11-rescued cells, we observed strong co-localization (merged yellow signals) between S100A11 and pan-Kla, confirming its robust lactylation in vivo. In contrast, in tumors expressing the K27R/K55R mutant, despite the strong accumulation of S100A11 protein (consistent with its stabilized state due to loss of ubiquitination), its co-localization with pan-Kla was profoundly diminished (Fig. 6D).

To further explore the clinical association of S100A11 lactylation with tumor progression, we re-analyzed a publicly available lactylome dataset [13] to compare the lactylation levels of S100A11 between gastric cancer patients at different TNM stages (*n* = 10). As shown in Supplementary Figure 4A-B, patients with advanced TNM stage (III/IV) exhibited consistently higher levels of S100A11 lactylation at both K27 and K55 sites, compared to patients with early stage (I/II). Although the difference did not reach statistical significance (likely due to the limited sample size of the cohort), this trend is consistent with our mechanistic findings that S100A11 lactylation promotes tumor progression and metastasis.

## Discussion

Metabolic reprogramming is a defining feature of the gastric cancer microenvironment, yet how the resultant accumulation of metabolites directly co-opts oncogenic protein networks remains incompletely understood [23, 24]. In this study, we delineate a previously unrecognized mechanism whereby lactate-driven lactylation of the oncoprotein S100A11 at K27 and K55 competitively blocks its FBXW11-mediated ubiquitination and proteasomal degradation, leading to protein stabilization, hyperactivation of PI3K-AKT and ERK1/2 signaling, and enhanced metastatic capacity. These findings establish a direct molecular link between the Warburg effect and the post-translational stabilization of a key oncogenic effector in gastric cancer.

A central advance of our work is the demonstration that lactylation and ubiquitination converge on the same lysine residues-K27 and K55-to dictate S100A11 protein fate. This competitive PTM crosstalk model is consistent with emerging paradigms in which acylation modifications, including acetylation and succinylation, antagonize ubiquitin-dependent degradation at shared lysine sites [25]. However, unlike acetylation, which is broadly regulated by diverse acetyltransferases, lactylation is uniquely coupled to glycolytic flux and lactate availability [4], positioning it as a metabolically responsive “switch” for protein stability. Thus, the lactylation–ubiquitination competition described here provides a mechanistic framework by which cancer cells can convert metabolic reprogramming into sustained oncogenic signaling. More broadly, this work suggests that metabolite-sensitive PTM crosstalk may represent a strategy through which tumors stabilize key oncogenic proteins under nutrient- and metabolism-altered conditions. Our observation that the p300 activator CTB and the HDAC inhibitor SAHA both augmented S100A11 lactylation suggests that the p300/HDAC axis serves as the enzymatic machinery governing this modification, in line with the reported roles of p300 as a lactyl-transferase and HDACs as potential delactylases [26]. Future studies employing catalytically inactive p300 mutants or isoform-specific HDAC knockdowns will be necessary to delineate the precise writers and erasers responsible for S100A11 site-specific lactylation.

The design of our K27R/K55R mutant merits careful interpretation. Replacing lysine with arginine renders these residues refractory to both lactylation and ubiquitination. Consequently, the K27R/K55R mutant phenocopies the stabilized state of lactylated S100A11—namely, the evasion of FBXW11-mediated degradation—without requiring lactate as a substrate. This explains the seemingly paradoxical observation that a “lactylation-deficient” mutant exhibits enhanced protein stability and augmented oncogenic signaling. The critical inference is that it is not lactylation per se that drives oncogenesis, but rather the shielding of K27 and K55 from ubiquitination, for which lactylation serves as the physiological mechanism within the lactate-rich tumor microenvironment. Crucially, our optimized biochemical assays elegantly uncoupled the processes of E3 ligase binding and ubiquitin conjugation. While physiological lactylation of wild-type S100A11 introduces bulky groups that sterically disrupt the S100A11–FBXW11 interaction, the K27R/K55R mutant remains fully accessible and constitutively binds FBXW11. However, because the exact lysine targets are missing, the bound E3 ligase is rendered futile, leading to enhanced stabilization of the unubiquitinated protein. This mechanism is further validated by the intermediate phenotypes of our individual single mutants (K27R and K55R), which maintain FBXW11 binding but still undergo partial degradation due to the ubiquitination of their remaining intact lysine residue.

It is worth noting that FBXW11 typically recognizes specific degron motifs rather than isolated lysine residues. Because our structural modeling confirmed that the overall protein architecture remains unaltered upon the K27R/K55R mutation (Supplementary Figure 2), the mechanism preventing FBXW11 binding is unlikely to be an artifact of protein misfolding. Instead, using lysine acetylation (Ac-K) as predictive proxy for acyl-modifications, our AlphaFold 3 model revealed that modifications at K27 and K55 introduce significant local steric hindrance and conformational flexibility. Given that lactyl groups are even bulkier, we propose that lactylation at these specific sites creates a robust spatial shield that physically masks the adjacent degron, thereby competitively obstructing FBXW11 recruitment.

Functionally, stabilized S100A11 potently amplifies PI3K-AKT and ERK1/2 signaling, two cascades with well-established roles in gastric cancer proliferation, survival, and metastasis [27, 28]. The concomitant upregulation of Cyclin D1 and c-Myc downstream of these pathways provides a mechanistic basis for the enhanced proliferative and metastatic phenotypes observed both in vitro and in our tail vein injection model. Notably, previous studies have attributed S100A11 oncogenic activity primarily to transcriptional upregulation [29, 30] or paracrine signaling [31, 32], Our findings extend this understanding by demonstrating that while transcriptional upregulation (as observed in our paired TCGA analysis) initiates *S100A11* expression, lactate-driven post-translational stabilization is a critical factor that further amplifies its protein abundance and functional impact. This synergistic regulation underscores the importance of investigating PTM-level control in cancer biology, as it explains how metabolic reprogramming directly reinforces oncogenic protein levels beyond transcriptional limits. Given that enhanced glycolysis and lactate accumulation are common features across many solid tumors [33], the mechanism identified here may not be restricted to gastric cancer. Instead, lactylation-mediated protection from ubiquitin-dependent degradation may represent a broader oncogenic principle by which lactate-rich tumors preserve metastasis-promoting proteins.

Several limitations of the present study should be acknowledged. First, our lactylome data were derived from a publicly available dataset with a limited sample size, and independent validation in larger, multi-center cohorts is warranted. Second, while we focused on K27 and K55 as the predominant lactylation sites, we cannot exclude the possibility that additional, lower-stoichiometry sites contribute to S100A11 regulation. Third, while our single-mutant rescue assays demonstrated the requirement of both K27 and K55 for ubiquitin conjugation, the K27R/K55R mutant cannot distinguish the loss of lactylation from the loss of ubiquitination at these lysine residues, because lysine-to-arginine substitution abolishes both modifications. Therefore, this mutant should be interpreted as a modification-refractory mutant. Future studies using site-specifically lactylated S100A11 proteins will be needed to directly dissect lactylation-dependent regulation from ubiquitination-site loss. Fourth, the tail vein injection model assesses the later steps of the metastatic cascade (extravasation and colonization) but does not recapitulate the full spectrum of spontaneous metastasis from a primary gastric tumor; orthotopic implantation models would provide more physiologically relevant validation. Finally, whether S100A11 lactylation influences additional aspects of the tumor microenvironment, such as immune cell recruitment or angiogenesis, remains to be explored. From a translational perspective, our findings highlight the lactate-S100A11 axis as a potential therapeutic vulnerability. A precise strategy might involve the development of customized molecular glues or PROTACs (PROteolysis Targeting Chimeras) specifically designed to bypass the lactylation shield and forcefully recruit E3 ligases to degrade S100A11.

In conclusion, this study reveals that the lactylation of S100A11 at K27 and K55, directly disrupts the ubiquitin-proteasome system to sustain oncogenic signaling, thereby establishing a mechanistic and potentially actionable link between gastric cancer metabolism and metastasis.

## Supplementary Information

Below is the link to the electronic supplementary material.

Supplementary Material 1

Supplementary Material 2

Supplementary Material 3

Supplementary Material 4

Supplementary Material 5

Supplementary Material 6

Supplementary Material 7

## Data Availability

Data will be made available on reasonable request.
