# Supplementary material for: Lactylation shields S100A11 from degradation to promote gastric cancer metastasis
Source: Cell Oncol (Dordr). 2026 Jul 9;49(5):130. doi: 10.1007/s13402-026-01255-w (PMC13631184; doi:10.1007/s13402-026-01255-w)

Figure 1G

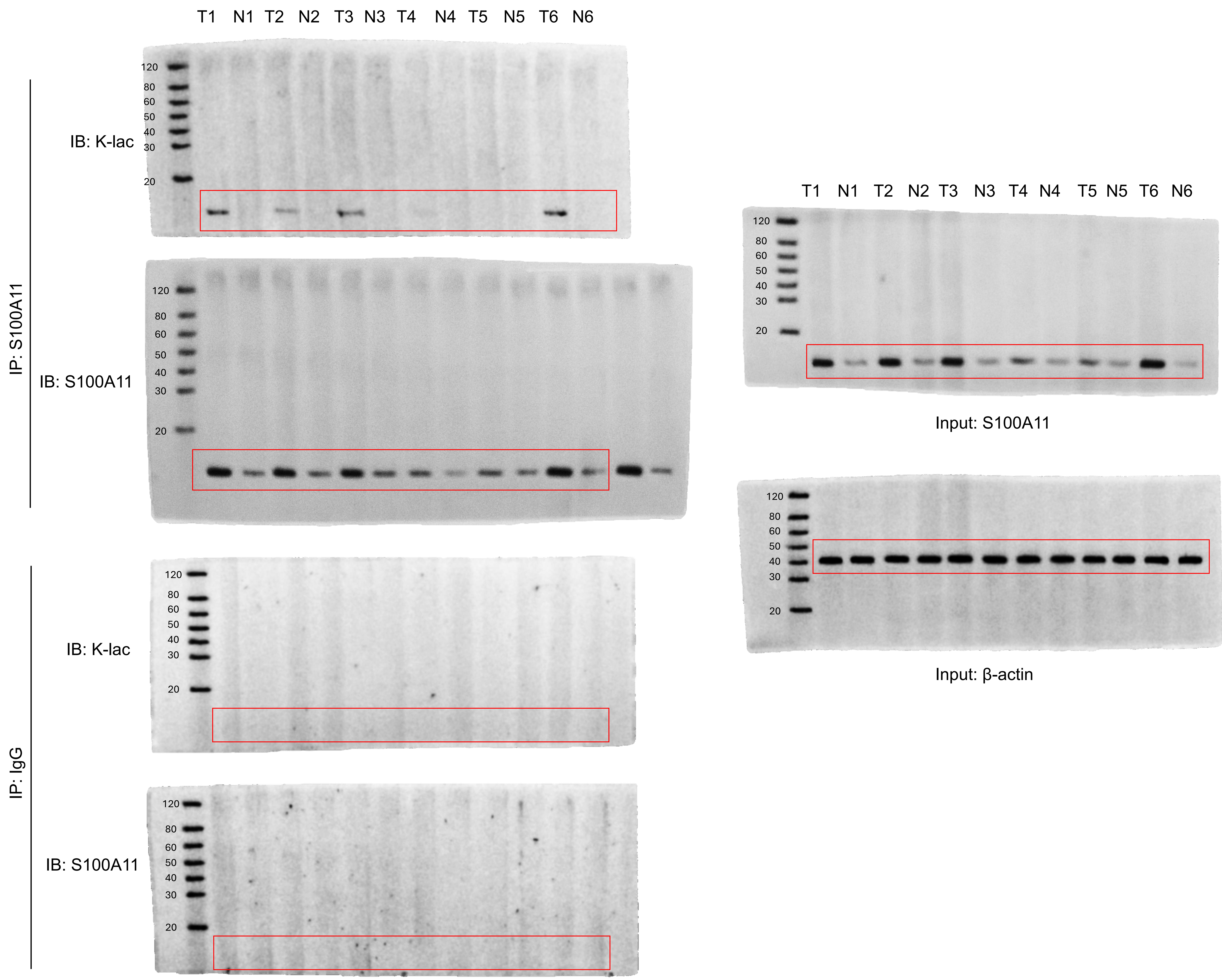

T1 N1 T2 N2 T3 N3 T4 N4 T5 N5 T6 N6
IP: S100A11
IB: K-lac
IB: S100A11
IP: IgG
IB: K-lac
IB: S100A11
120
80
60
50
40
30
20
Input: S100A11
Input: β-actin

Figure 2A

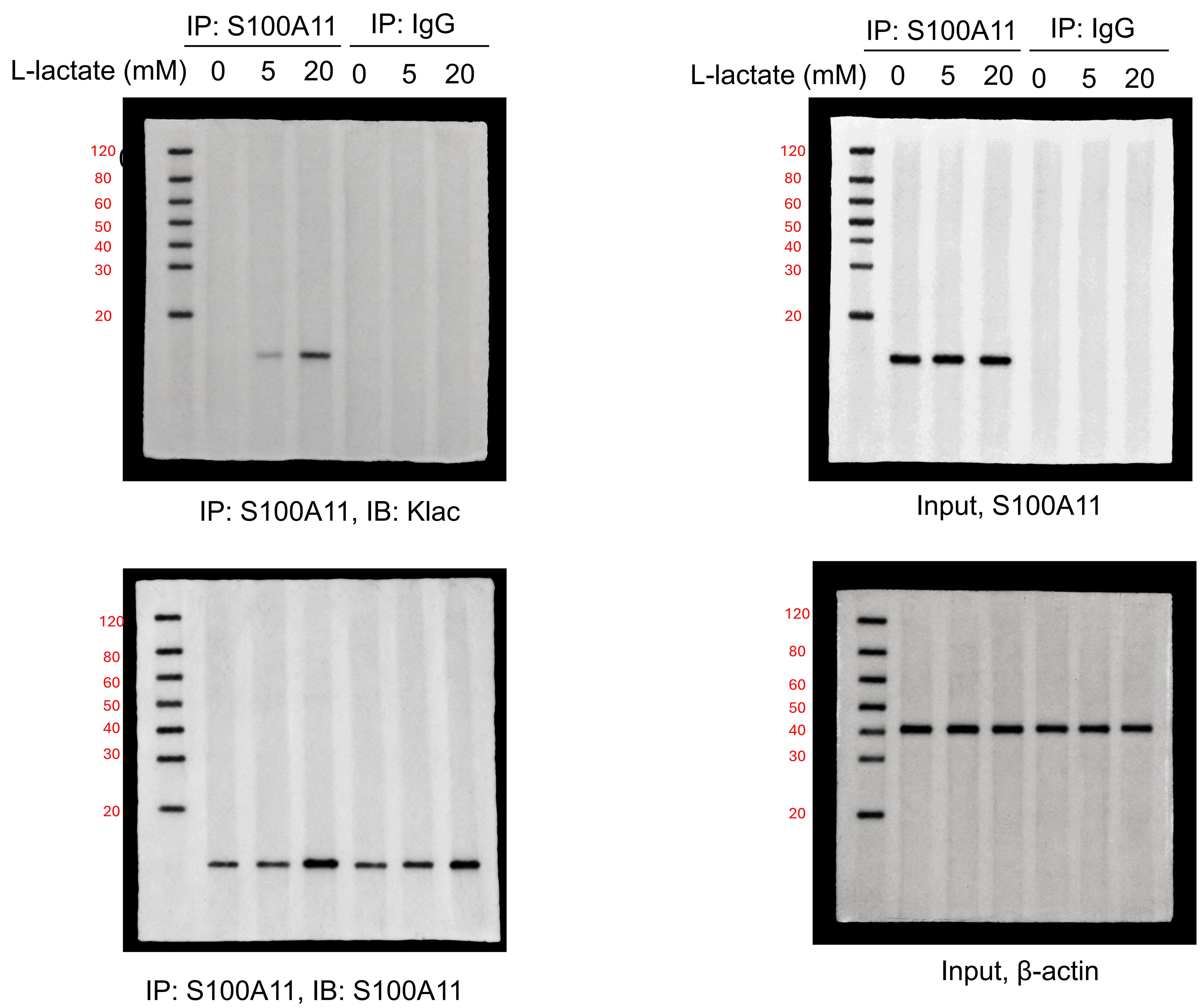

AGS

Figure 2B

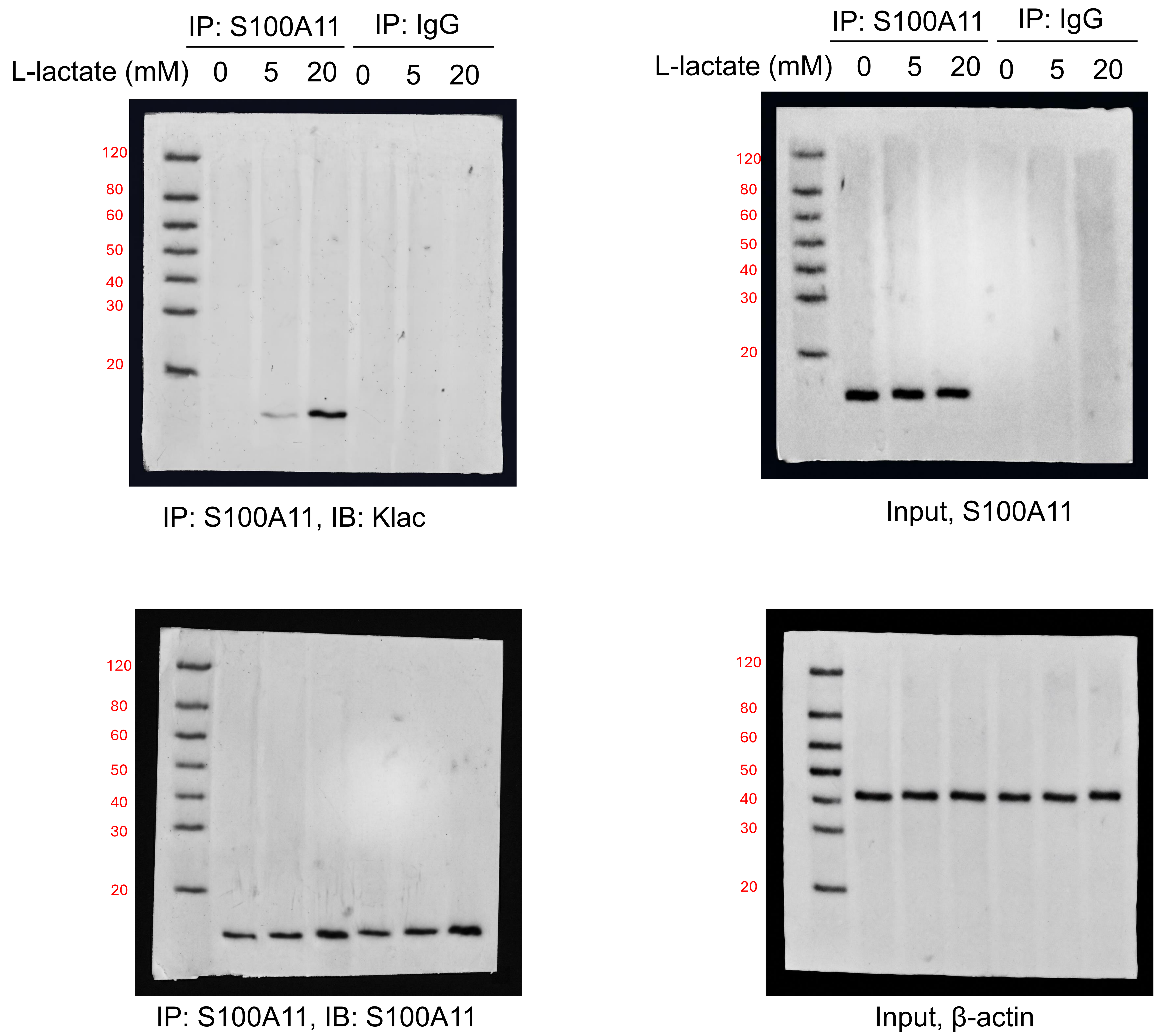

MKN-45

Figure 2C-D

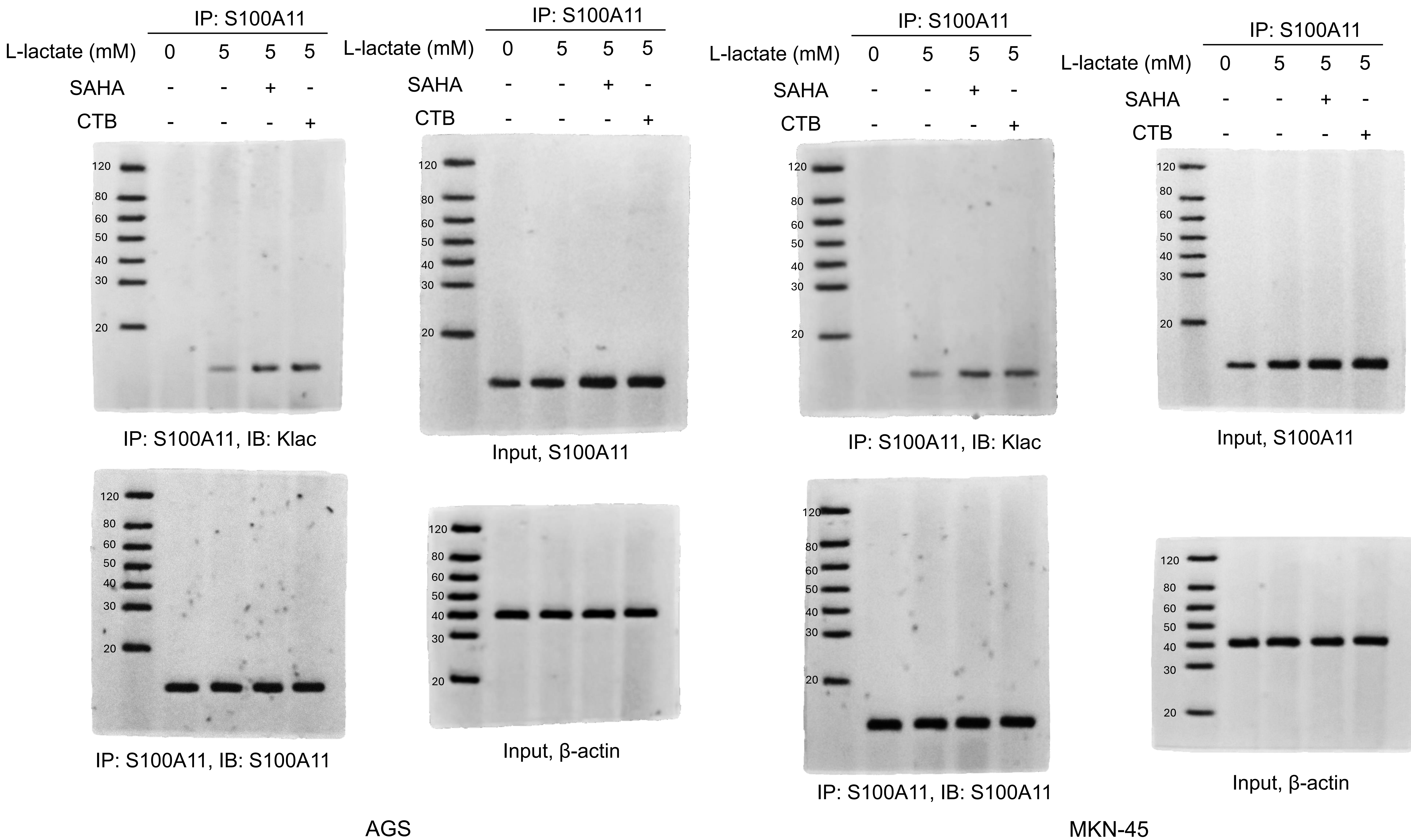
IP: S100A11
L-lactate (mM) 0 5 5 5
SAHA - - + -
CTB - - - +
120
80
60
50
40
30
20
IP: S100A11, IB: Klac
Input, S100A11
IP: S100A11, IB: S100A11
Input, β-actin
AGS
MKN-45

Figure 2E-F

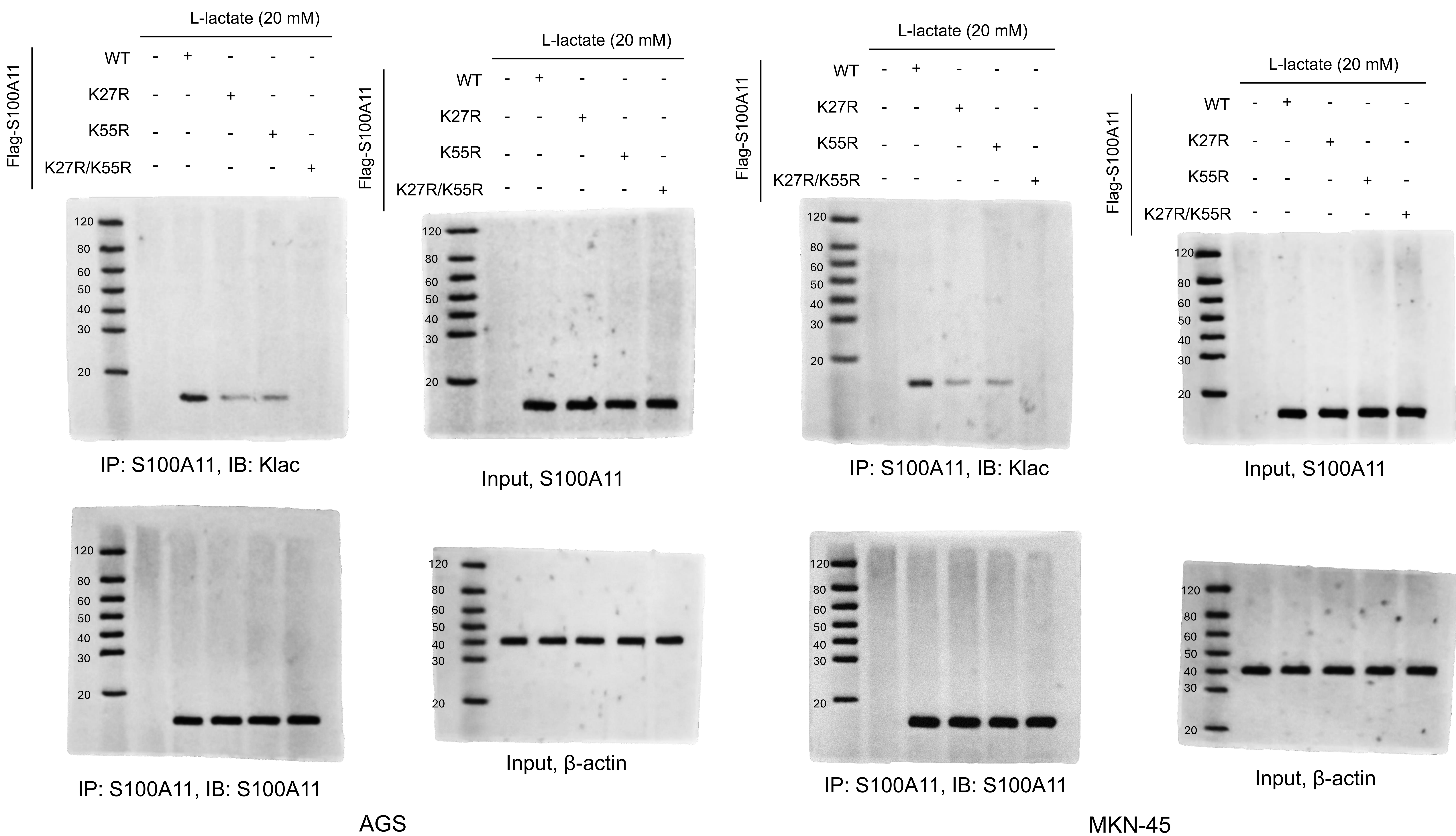
L-lactate (20 mM)
Flag-S100A11
WT
K27R
K55R
K27R/K55R
IP: S100A11, IB: Klac
Input, S100A11
IP: S100A11, IB: S100A11
Input, β-actin
AGS
MKN-45

Figure 3A

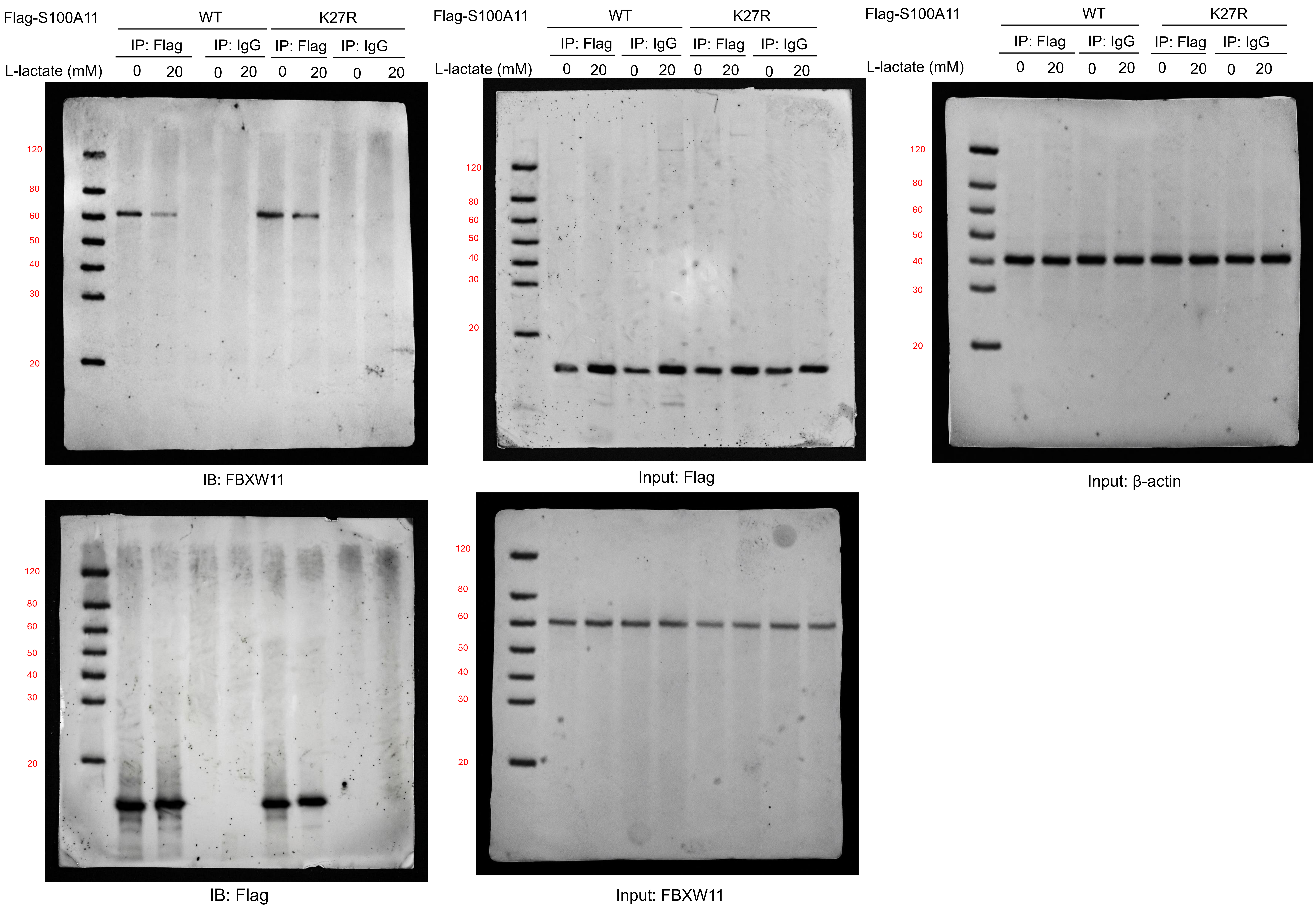

AGS

Figure 3B

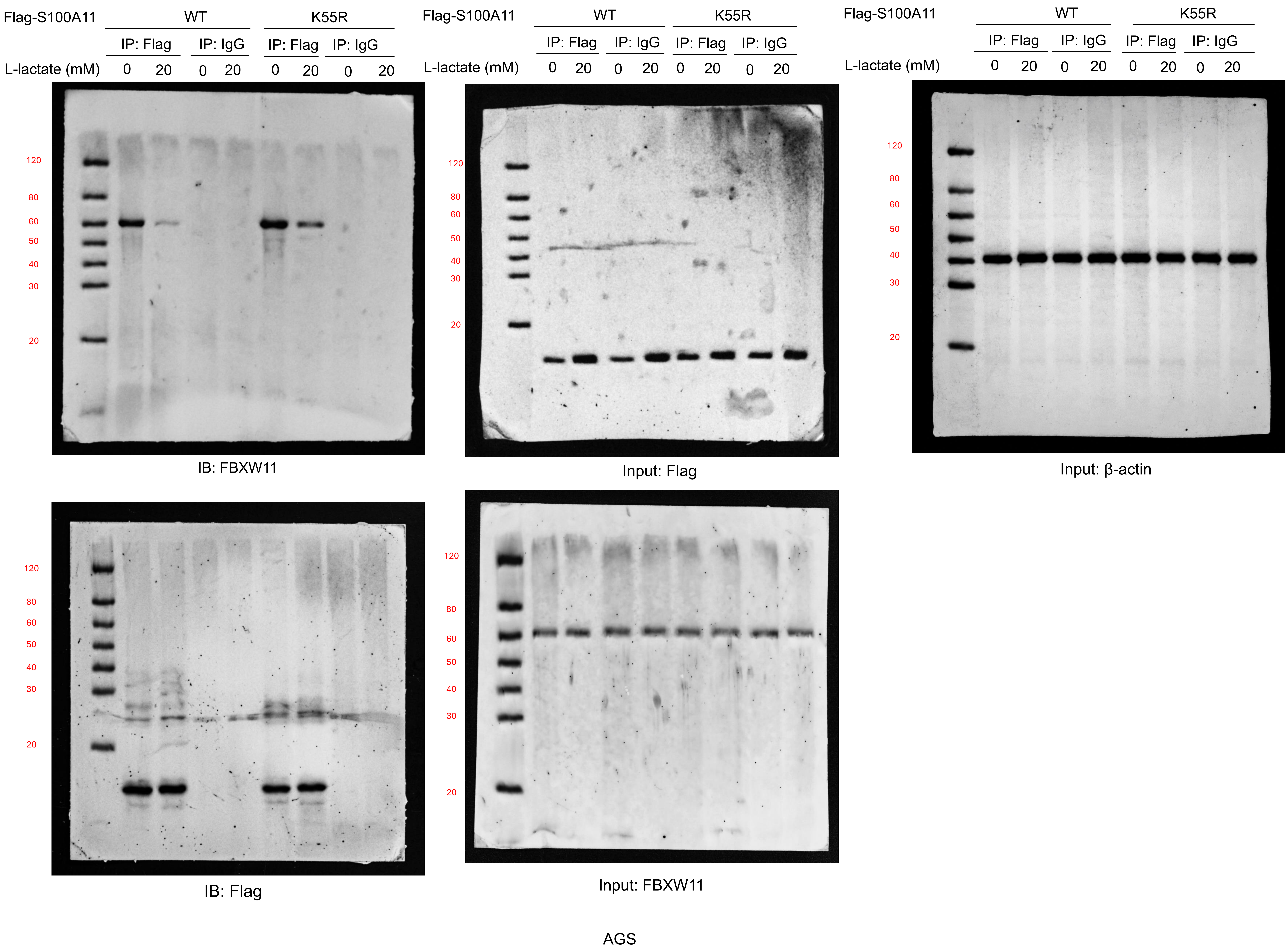
Flag-S100A11
WT
K55R
IP: Flag
IP: IgG
L-lactate (mM)
0
20
120
80
60
50
40
30
20
IB: FBXW11
IB: Flag
Input: Flag
Input: FBXW11
Input: β-actin
AGS

Figure 3C

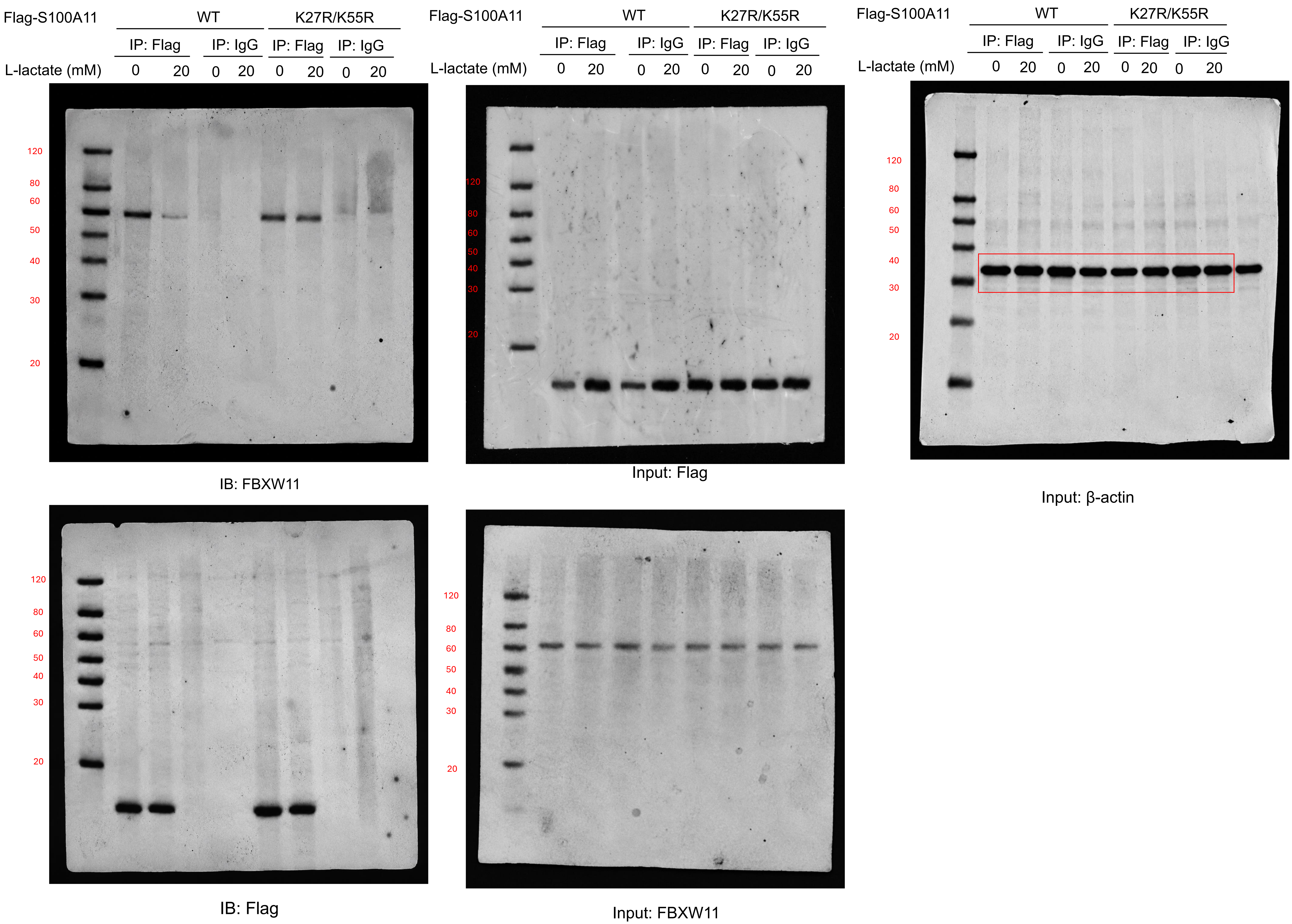

AGS

Figure 3D-E

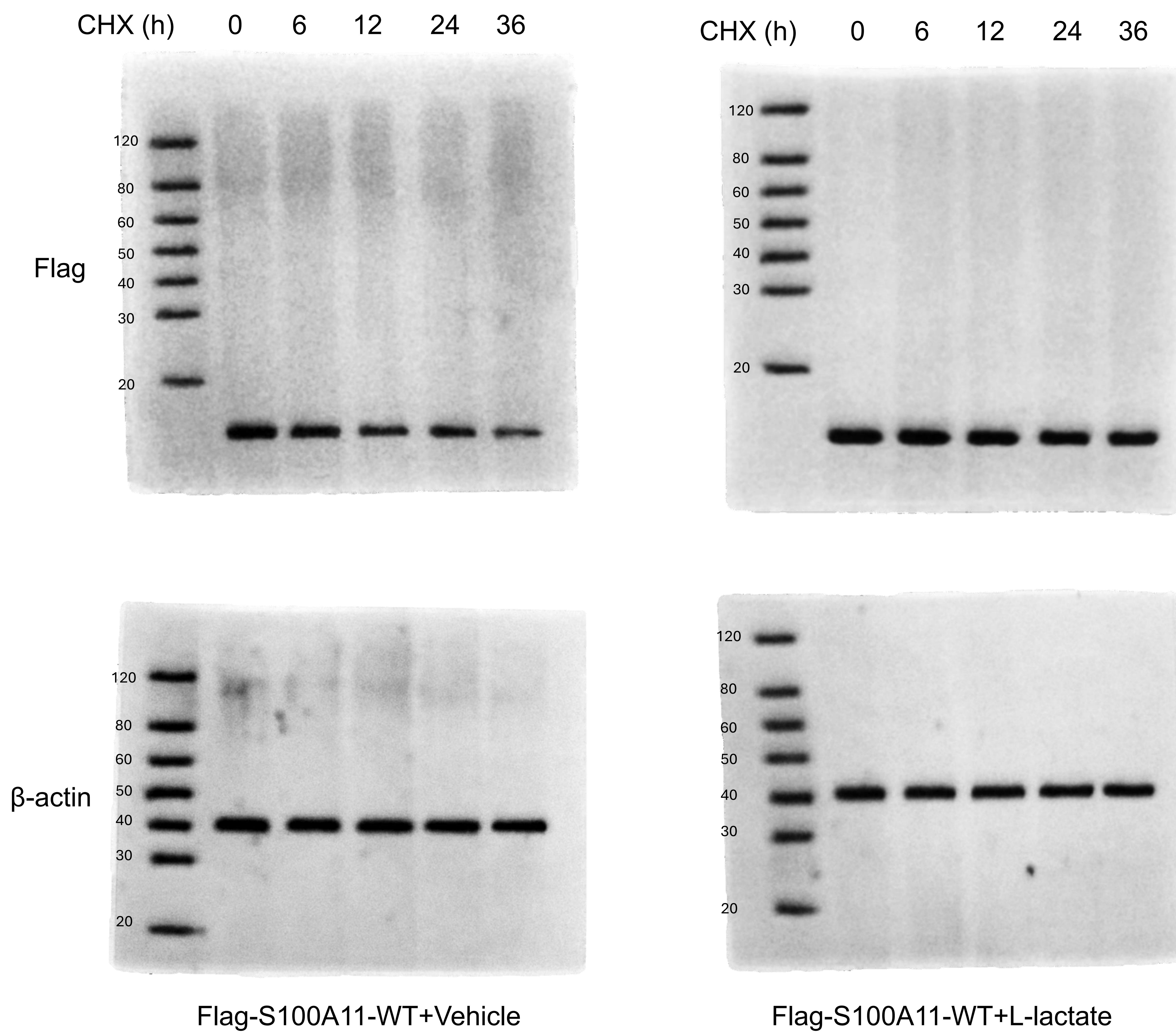
CHX (h)
0
6
12
24
36
Flag
β-actin
120
80
60
50
40
30
20
Flag-S100A11-WT+Vehicle
Flag-S100A11-WT+L-lactate

Figure 3F-G

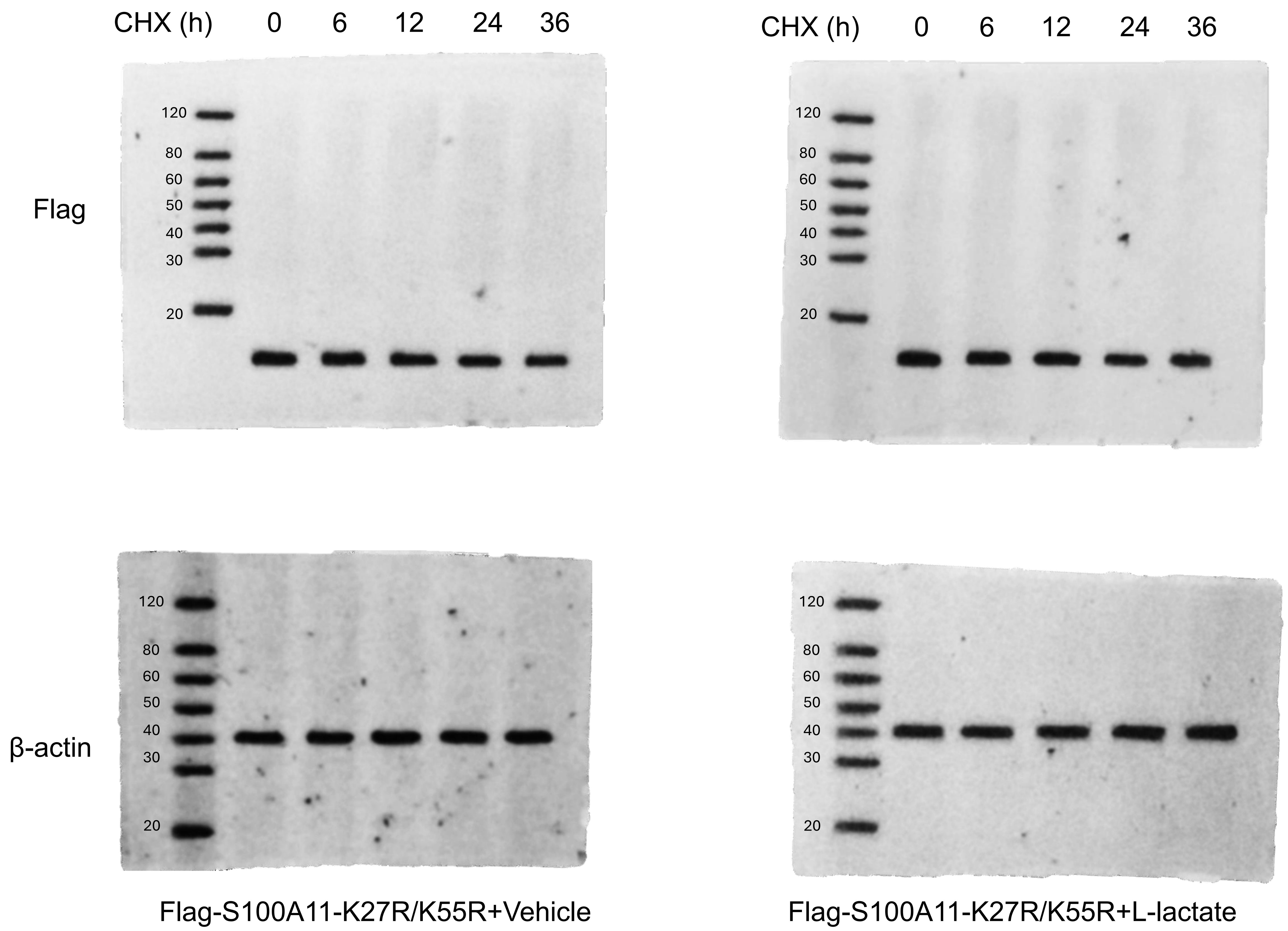

CHX (h)
0
6
12
24
36
Flag
β-actin
120
80
60
50
40
30
20
Flag-S100A11-K27R/K55R+Vehicle
Flag-S100A11-K27R/K55R+L-lactate

Figure 3K

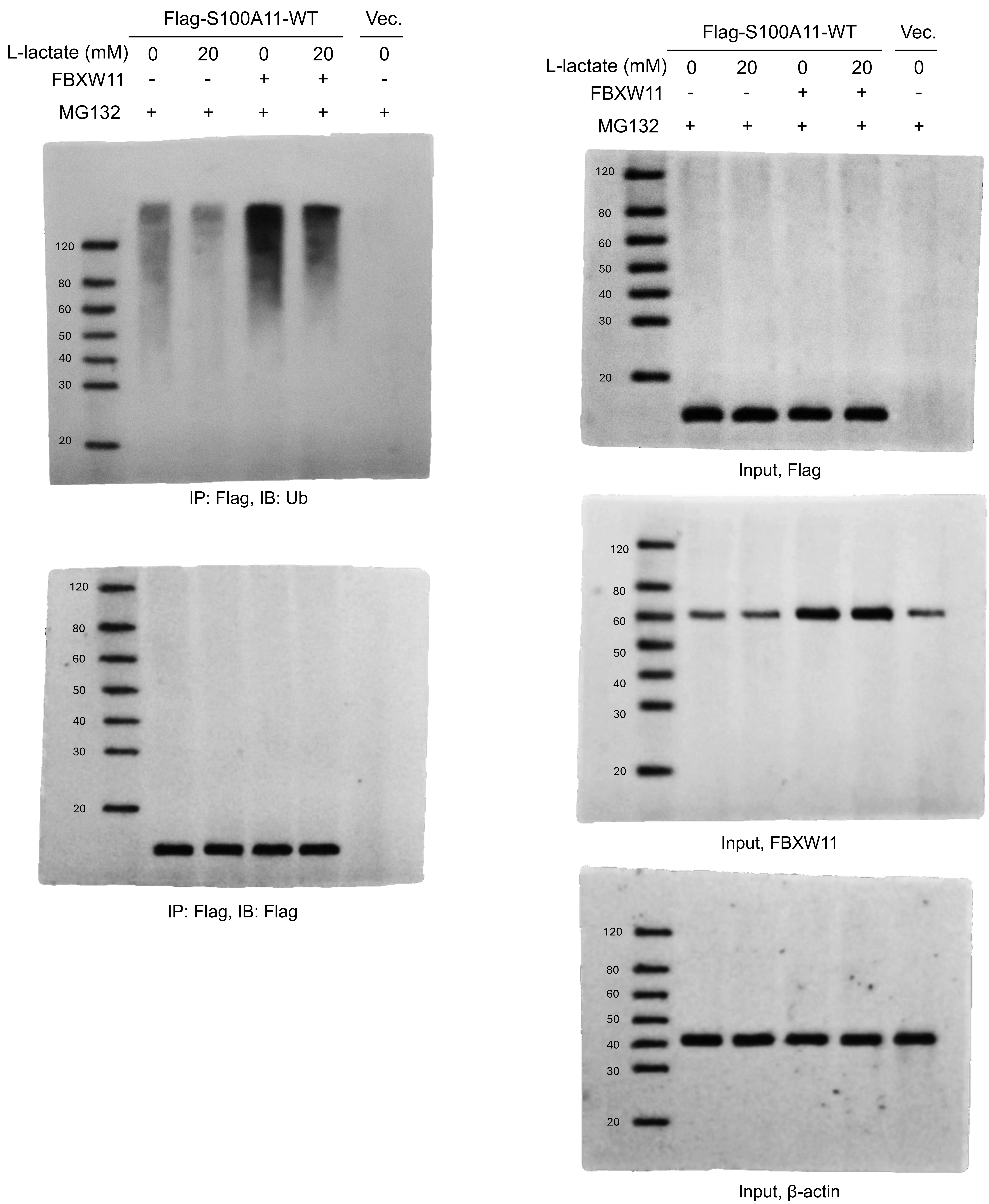

AGS

Figure 3L

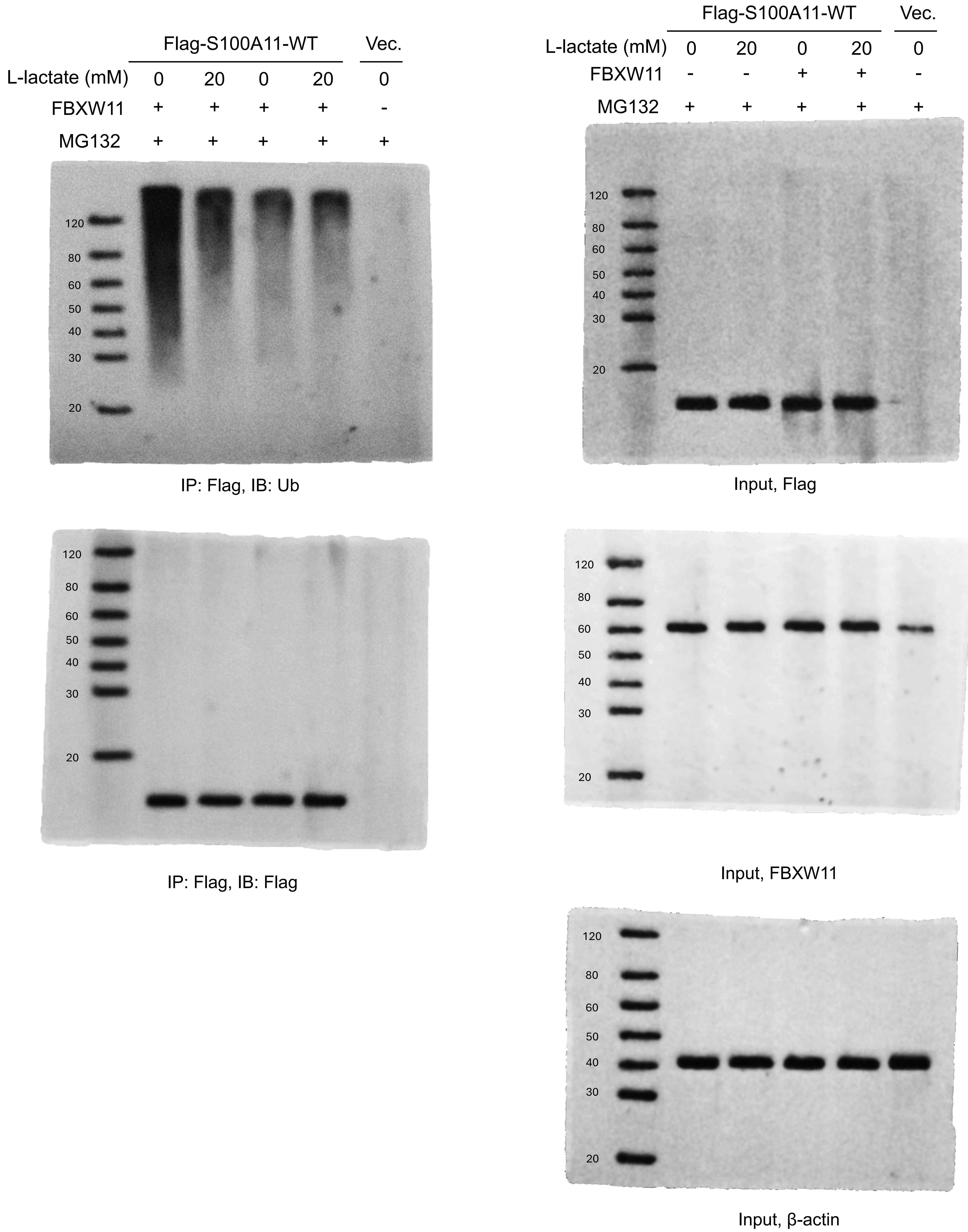

MKN-45

Figure 4A

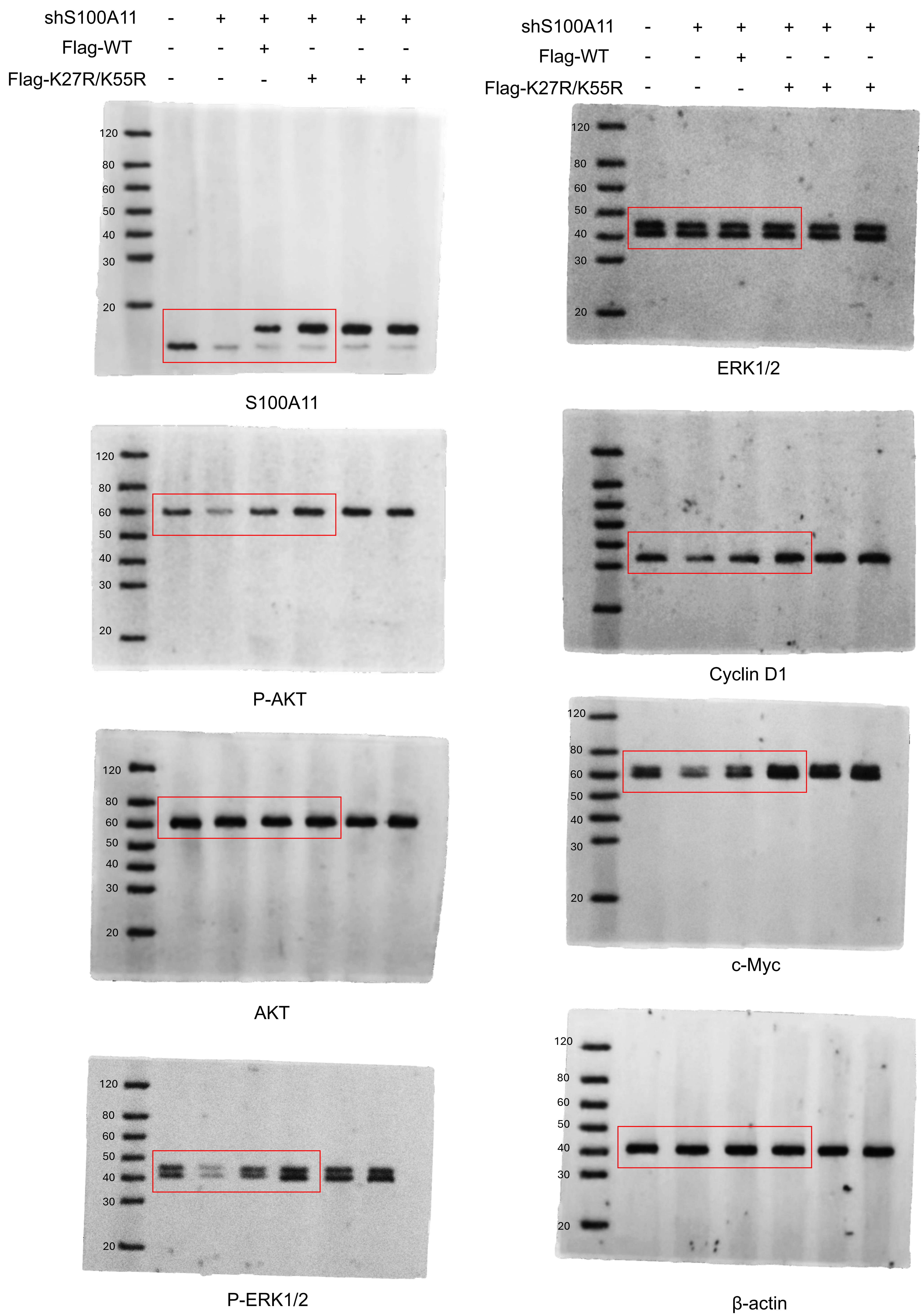
shS100A11 - + + + + +
Flag-WT - - + - - -
Flag-K27R/K55R - - - + + +
shS100A11 - + + + + +
Flag-WT - - + - - -
Flag-K27R/K55R - - - + + +
120
80
60
50
40
30
20
S100A11
ERK1/2
P-AKT
Cyclin D1
AKT
c-Myc
P-ERK1/2
β-actin

Figure 4C

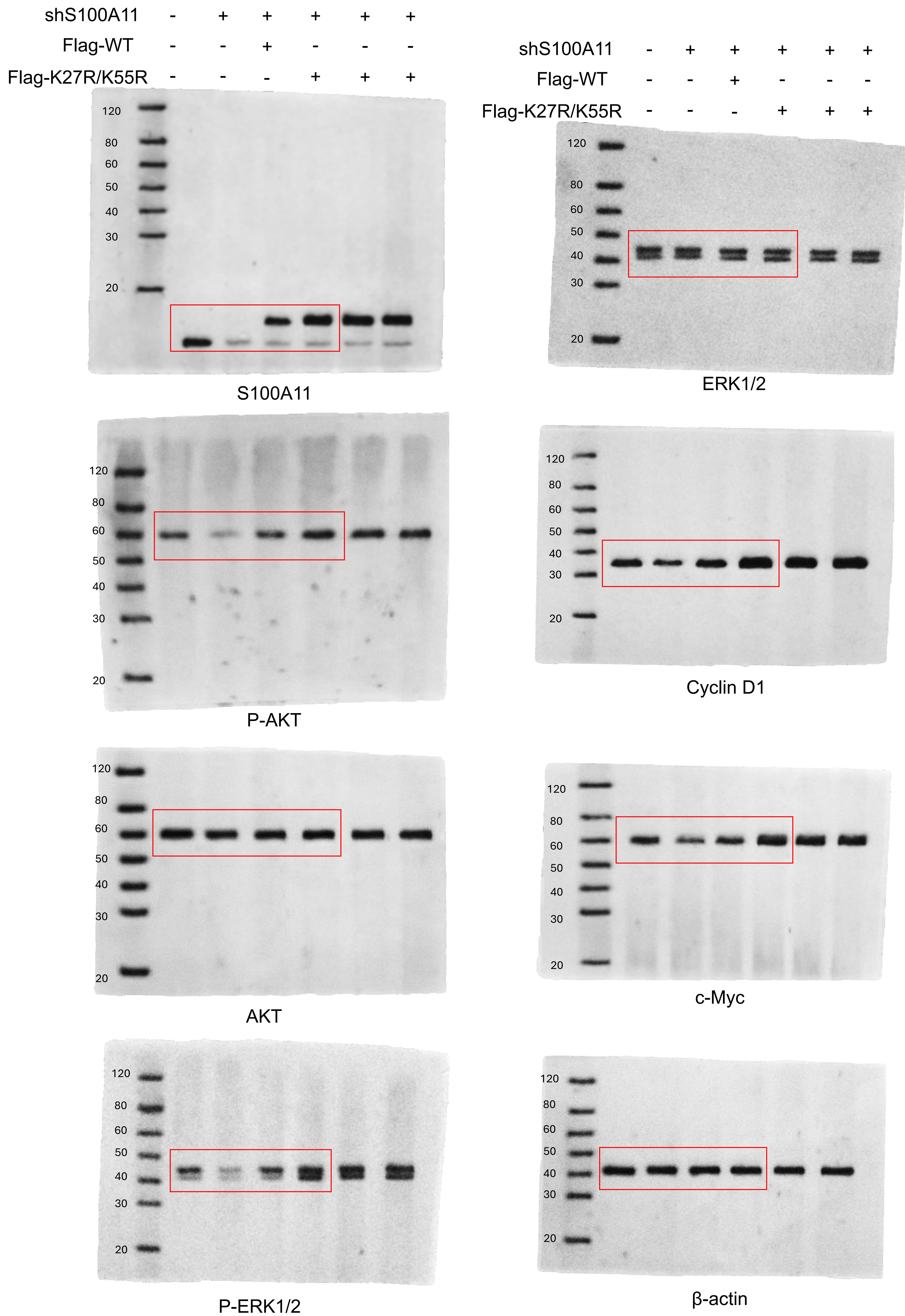

MKN-45

Supplementary Figure 3A

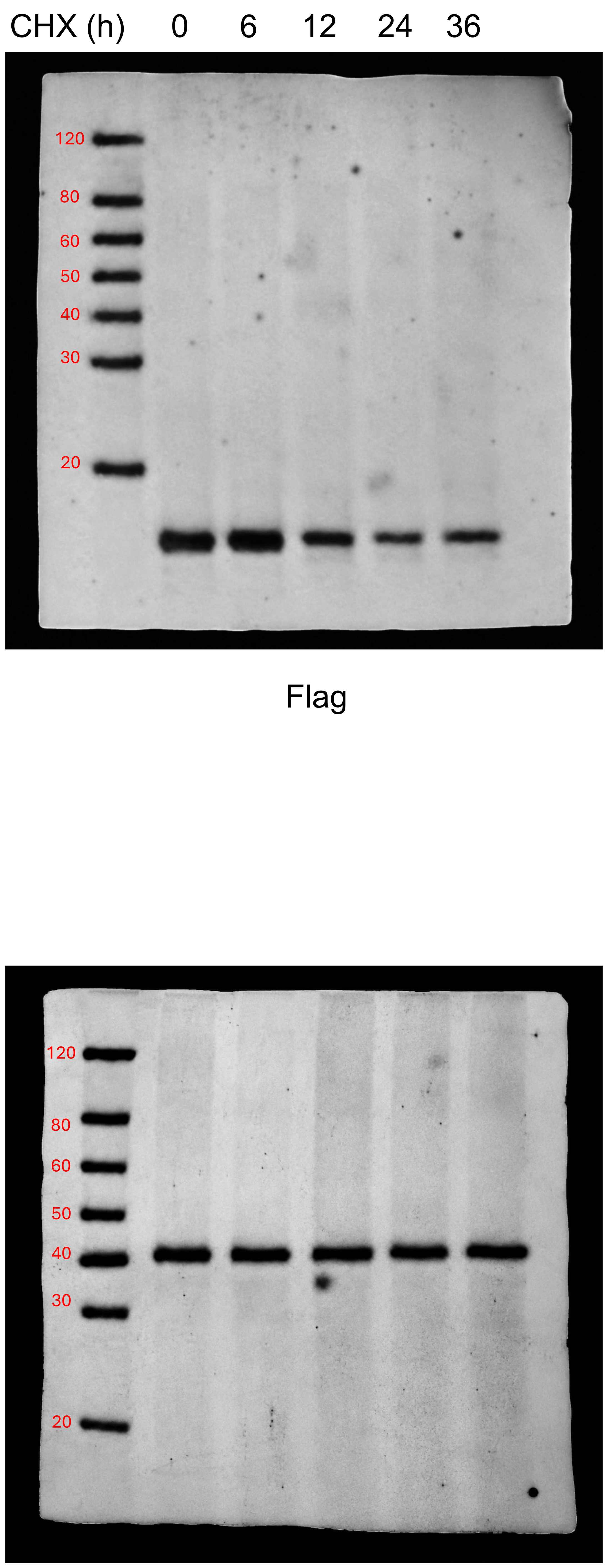

Supplementary Figure 3B

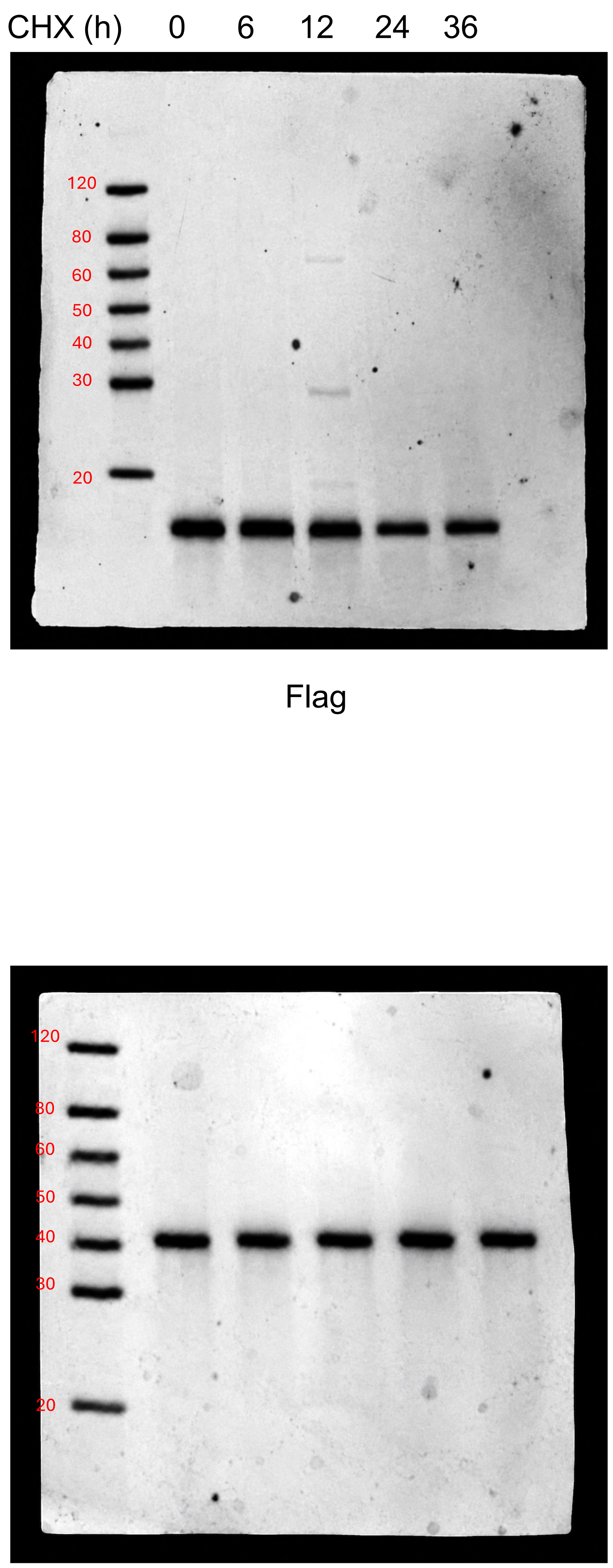

Supplementary Figure 3D

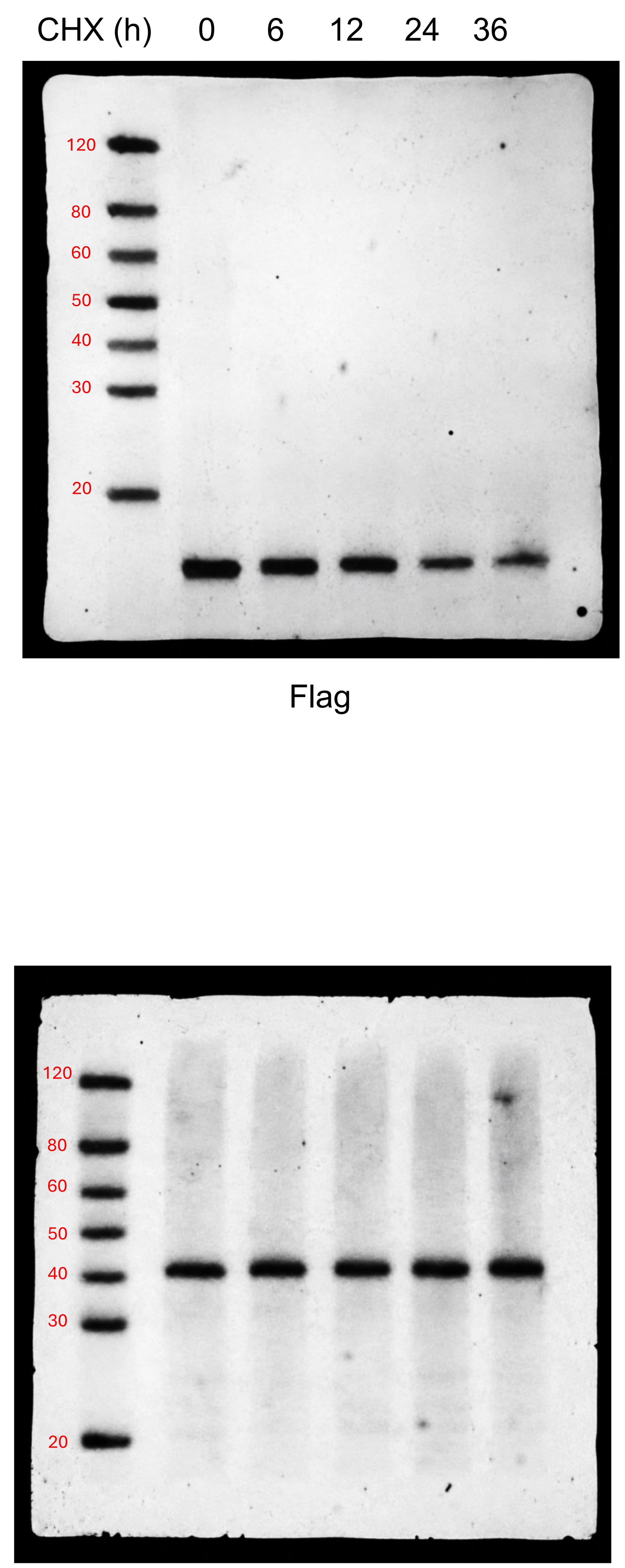

Supplementary Figure 3E

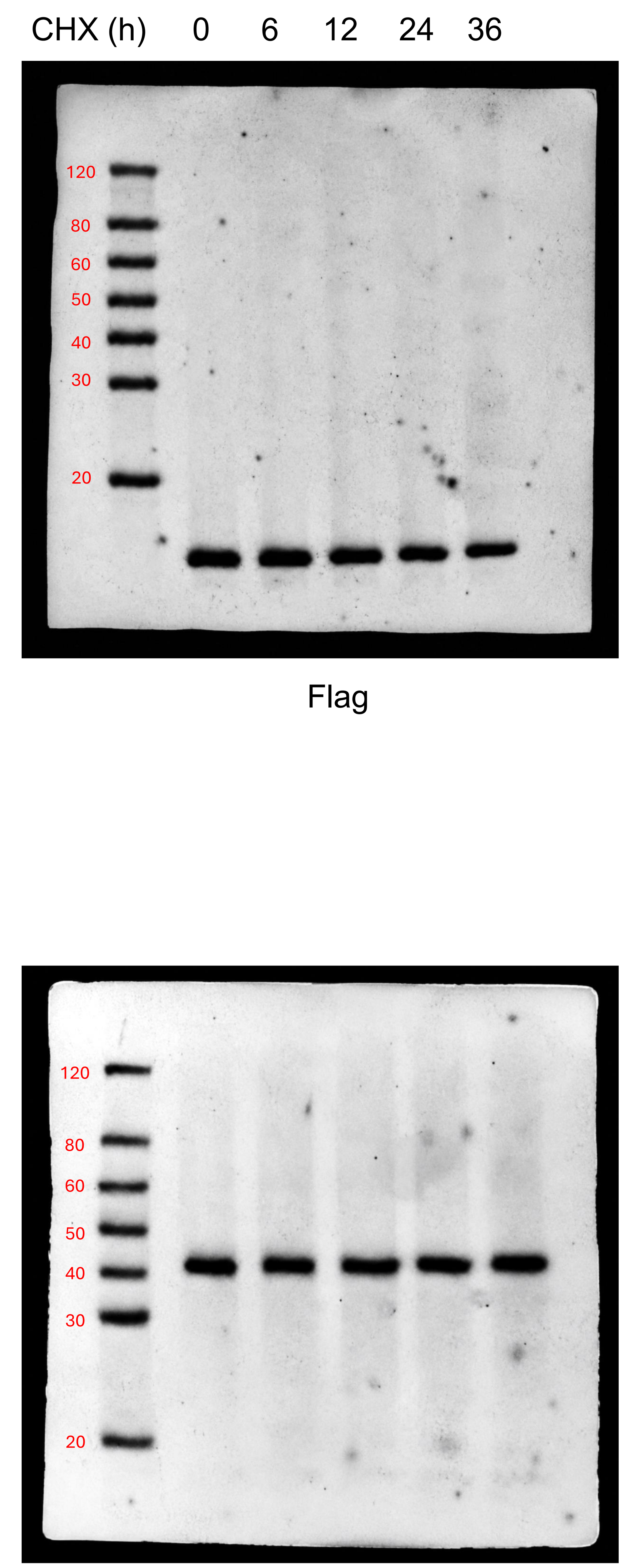

Supplement: Supplementary file 1 [file 13402_2026_1255_MOESM5_ESM.pdf]
