# Supplementary material for: Lactylation shields S100A11 from degradation to promote gastric cancer metastasis
Source: Cell Oncol (Dordr). 2026 Jul 9;49(5):130. doi: 10.1007/s13402-026-01255-w (PMC13631184; doi:10.1007/s13402-026-01255-w)
Supplement: Supplementary file 3 [file 13402_2026_1255_MOESM7_ESM.docx]

**Materials and Methods**

**Gastric tissue collection and processing**
Lactylation data integration and identification of differentially lactylated S100 proteins were performed using the data from a recent publication ^[1]^. In this dataset, primary gastric cancer tissues and matched adjacent normal tissues were collected from patients undergoing surgical resection (n=10 per group). Tissue samples were enzymatically digested and subjected to immunoprecipitation using anti-lactyllysine antibodies. The enriched proteins were analyzed by liquid chromatography-tandem mass spectrometry (LC-MS/MS).

**Bioinformatic analysis**
Differential lactylation was assessed using normalized K-lac intensity values. Gene expression profiles of S100 family members were examined using RNA-seq data from TCGA-STAD ^[2]^, using access provided the UCSC Xena Browser ^[3]^. Survival analysis for S100A family gene expression was performed using the Kaplan-Meier plotter database ^[4]^, with high/low expression groups stratified by median expression values and significance assessed via log-rank test (*p* < 0.05 considered significant). Protein expression validation was conducted using the Human Protein Atlas (https://www.proteinatlas.org/) ^[5,6]^. S100A11 protein expression in stomach cancer and adjacent normal tissues were retrieved from the datasets of the National Cancer Institute’s Clinical Proteomic Tumor Analysis Consortium (CPTAC), by using The Cancer Proteogenomic Data Analysis Site (cProSite) ^[7]^.

**Cell Culture and Treatments**
Human gastric cancer cell lines AGS and MKN-45 were obtained from the Procell (Wuhan, China) and cultured in RPMI-1640 medium supplemented with 10% fetal bovine serum (FBS) at 37°C in a humidified atmosphere containing 5% CO₂. For lactate treatment, cells were incubated with 0, 5, or 20 mM sodium L-lactate (Sigma-Aldrich) for 24 h. Where indicated, cells were treated with 5 mM L-lactate in the presence or absence of 5 μM suberoylanilide hydroxamic acid (SAHA, a pan-HDAC inhibitor, Selleck) or 10 μM p300 activator CTB (HY-134964 ,MCE) for 24 h.

**Plasmid construction and infection**
Wild-type human S100A11 cDNA was cloned into the pLVpuro-CMV-N-3xFLAG lentiviral transfer vector. The K27R, K55R, and the double mutant (K27R/K55R) were generated via chemical synthesis of the respective cDNA sequences (General Biosystems, China) and subsequently cloned into the same lentiviral vector. For knockdown experiments, shRNA oligonucleotides targeting human *S100A11* (shS100A11, 5’-CGGTGCATCGAGTCCCTGATT-3’) and a non-targeting control (shNC) were synthesized by General Biosystems and cloned into a lentiviral vector carrying a hygromycin resistance gene (pLKO.1-hygro) to allow for dual antibiotic selection during the subsequent rescue experiments.

Lentiviral particles were produced by co-transfecting HEK293T cells with the transfer plasmid, packaging plasmid (psPAX2), and envelope plasmid (pMD2.G) using Lipofectamine 3000 (Invitrogen) according to the manufacturer’s instructions. Viral supernatants were collected 48-72 h post-transfection, filtered through a 0.45 μm filter, and concentrated using ultracentrifugation or Lenti-X concentrator (Takara Bio).

To generate the knockdown and rescue cell lines, AGS and MKN-45 cells were first transduced with shNC or shS100A11 lentiviral particles at a multiplicity of infection (MOI) of 5 in the presence of 8 μg/mL polybrene. After 48 h, cells were selected with 200 μg/mL hygromycin for 5 days to establish stable knockdown lines. Subsequently, the shS100A11-expressing cells were transduced with the puromycin-resistant lentiviruses encoding Flag-tagged S100A11-WT or the K27R/K55R mutant. After 48 h, these dual-infected cells were subjected to a second selection step using 2 μg/mL puromycin for 3 days. Expression and knockdown efficiency of Flag-tagged S100A11 (wild-type or mutants) were confirmed by western blotting prior to experiments.

**Immunoprecipitation and western blot**

**Primary gastric cancer tissues were collected from patients undergoing surgical resection at China-Japan Friendship Hospital, with approval from the institutional review board (IRB approval number: 2023-KY-174) and informed consent obtained from all participants (n=6 cases, age range 45-72 years, stages II-III adenocarcinoma). Tissues were snap-frozen in liquid nitrogen and stored at -80°C until use. Tumor adjacent normal tissues were used as controls. For sample preparation, tissue samples were homogenized, and both tissues and cultured cells were lysed in IP lysis buffer (Beyotime) supplemented with a protease inhibitor cocktail.** **To specifically capture the transient interaction between S100A11 and the E3 ligase FBXW11, a mild formulation of the IP lysis buffer (containing 1% NP-40) was utilized, and the proteasome inhibitor MG132 (10 μM) was added to the lysis buffer to prevent the rapid degradation of unprotected substrate proteins during extraction.**

**For the immunoprecipitation of endogenous S100A11, equal amounts of tissue or cell lysates were incubated with an anti-S100A11 antibody (rabbit polyclonal, 10237-1-AP, Proteintech) or control IgG overnight at 4°C, followed by the addition of Protein A/G magnetic beads (Thermo Fisher Scientific) for 2 h at room temperature. For the immunoprecipitation of Flag-tagged proteins, cell lysates were incubated directly with anti-Flag M2 magnetic beads (Sigma-Aldrich) overnight at 4°C. Following incubation, the magnetic beads were isolated using a magnetic separation rack and washed three times with a mild, low-detergent cold wash buffer to remove non-specifically bound proteins while minimizing the dissociation of transient E3 ligase complexes.**

**The resulting immunoprecipitates and total input lysates were boiled in SDS sample buffer,** resolved by SDS-PAGE, and transferred to PVDF membranes. Membranes were probed with anti-lactyllysine (K-lac, mouse monoclonal, KC-2333, Absea), anti-S100A11 (mouse monoclonal, 60024-1-Ig, Proteintech), anti-FBXW11 (mouse monoclonal, 68090-1-Ig, Proteintech), anti-Flag (mouse monoclonal, 66008-4-Ig, Proteintech), and anti-β-actin (mouse monoclonal, 66009-1-Ig, Proteintech) antibodies.

Lysates from primary gastric cancer tissues were immunoprecipitated with anti-S100A11 (rabbit polyclonal, 1:1000, 10237-1-AP, Proteintech) or IgG control antibodies. Western blotting was performed to detect lactylation (K-lac) (mouse monoclonal, 1:1000, KC-2333, Absea) and S100A11 (mouse monoclonal, 1:1000, 60024-1-Ig, Proteintech) levels.

For signaling detection related western blotting, the following antibodies were used:   **anti-phospho-AKT (Ser473, 4060, CST), anti-AKT (9272, CST), anti-phospho-ERK1/2 (Thr202/Tyr204, 4370, CST), anti-ERK1/2 (4695, CST), anti-Cyclin D1 (60186-1-Ig, Proteintech), anti-c-Myc (10828-1-AP, Proteintech).**

***In vivo* endogenous ubiquitination assay**

AGS cells stably expressing Flag-S100A11-WT or K27R/K55R were treated with or without 20 mM L-lactate for 24 h. To accumulate ubiquitinated proteins, cells were treated with 10 μM of the proteasome inhibitor MG132 (Sigma-Aldrich) for 6 h prior to harvest. Cells were lysed in denaturing buffer (1% SDS, 50 mM Tris-HCl pH 7.5, 150 mM NaCl, 1 mM EDTA, 10 mM N-ethylmaleimide [NEM, to inhibit deubiquitinases], and protease inhibitors) and boiled at 95°C for 10 min to disrupt non-covalent protein-protein interactions. The lysates were then diluted 10-fold with standard IP lysis buffer to reduce the SDS concentration to 0.1%. Lysates were immunoprecipitated with anti-Flag M2 magnetic beads overnight at 4°C. Endogenous ubiquitination of S100A11 was detected by immunoblotting with an anti-ubiquitin antibody (rabbit polyclonal, 10201-2-AP, Proteintech) and anti-Flag (rabbit monoclonal, 80801-2-RR, Proteintech) antibodies.

**Cycloheximide (CHX) chase assay**
AGS cells stably expressing Flag-S100A11-WT or K27R/K55R were pretreated with or without 20 mM L-lactate for 24 h, then incubated with 100 μg/mL cycloheximide (CHX, Sigma-Aldrich) for the indicated times (0, 6, 12, 24, 36 h). Cells were harvested at each time point, and Flag-S100A11 protein levels were analyzed by western blotting.

**Cell proliferation assay (CCK-8)
Cell proliferation was assessed using the Cell Counting Kit-8 (CCK-8, Dojindo). AGS and MKN-45 cells with S100A11 knockdown and rescue by wild-type or K27R/K55R mutant S100A11 were seeded in 96-well plates at 2,000 cells/well. At 0, 24, 48, and 72 h, 10 μL of CCK-8 reagent was added to each well and incubated for 2 h at 37°C. Absorbance at 450 nm was measured using a microplate reader.**

**Colony formation assay
For colony formation, 500 cells per well were seeded in 6-well plates and cultured for 10–14 days. Colonies were fixed with 4% paraformaldehyde and stained with 0.1% crystal violet. Colonies containing more than 50 cells were counted under a microscope.**

**Transwell migration and invasion assays
Cell migration and invasion were evaluated using 24-well transwell chambers (8 μm pore size, Corning). For migration assays, 5 × 10⁴ cells in serum-free medium were seeded in the upper chamber. For invasion assays, the upper chamber was pre-coated with Matrigel (BD Biosciences), and 1 × 10⁵ cells were seeded. Medium containing 10% FBS was added to the lower chamber as a chemoattractant. After 24 h incubation, cells on the upper surface were removed, and migrated/invaded cells on the lower surface were fixed, stained with crystal violet, and counted in five random fields per well under a microscope.**

***In vivo* metastasis assay**

**To assess the effect of S100A11 lactylation on gastric cancer metastasis *in vivo*, a tail vein injection model was established. AGS cells with stable S100A11 knockdown (shS100A11) and rescue by wild-type (WT) or K27R/K55R mutant S100A11 were prepared as described above. A total of 5 × 10^5^ cells in 100 μL PBS were injected into the lateral tail vein of 6–8-week-old female BALB/c nude mice (Beijing Vital River Laboratory Animal Technology Co., Ltd., license number: SYXK (Jing) 2023-0012). Mice were randomly divided into four groups (shNC, shS100A11, shS100A11+WT, shS100A11+K27R/K55R), with 8 mice per group. Mice were monitored daily for general health and body weight. After 21 days, mice were euthanized, and lungs were harvested, fixed in 4% paraformaldehyde, embedded in paraffin, and sectioned for hematoxylin and eosin (H&E) staining. Metastatic tumor burden was quantified as the percentage of metastatic area per lung section using ImageJ software. All animal procedures were approved by the Institutional Animal Care and Use Committee. Animal welfare and humane endpoints were strictly observed. Mice were housed in SPF conditions (22–25°C, 50–60% humidity, 12 h light/dark cycle), with ad libitum access to food and water. Humane endpoints included >15% body weight loss, severe respiratory distress, or moribund state, at which point mice were euthanized immediately.**

**Multiplex immunofluorescence (mIHC)**

For the mIHC staining of mouse lung tissue sections, paraffin-embedded sections were first deparaffinized in xylene and rehydrated through a graded series of ethanol. Antigen retrieval was performed by heating the sections in 10 mM citrate buffer (pH 6.0) at 95°C for 20 min. After cooling to room temperature, sections were permeabilized with 0.3% Triton X-100 in PBS for 15 min, followed by blocking with 5% BSA in PBS for 1 h at room temperature to prevent non-specific antibody binding.

Sections were then incubated overnight at 4°C with primary antibodies diluted in blocking buffer: rabbit anti-S100A11 (1:200, 10237-1-AP, Proteintech) and mouse anti-pan-lactyllysine (Pan-Kla, 1:200, KC-2333, Absea). After washing three times with PBS, sections were incubated for 1 h at room temperature with the corresponding fluorophore-conjugated secondary antibodies: Alexa Fluor 488-conjugated goat anti-rabbit IgG and Alexa Fluor 594-conjugated goat anti-mouse IgG (Thermo Fisher Scientific), diluted 1:500 in blocking buffer. Nuclei were counterstained with DAPI (4',6-diamidino-2-phenylindole) for 5 min to visualize the nuclear structure. Finally, sections were mounted with anti-fade mounting medium (Vector Laboratories) to prevent fluorescence quenching. Fluorescence images were acquired using a confocal laser scanning microscope (Leica TCS SP8).

**Statistical analysis**
Statistical analyses were performed using GraphPad Prism software (GraphPad 10.1.6, Inc., San Diego, CA, USA). All quantitative data are presented as the mean ± standard deviation (SD) from at least three independent in vitro experiments, unless otherwise stated. For comparisons between two independent groups, a two-tailed Student’s t-test was utilized. For comparisons involving three or more groups, a one-way analysis of variance (ANOVA) followed by Tukey’s post hoc test was applied. For survival analysis, curves were generated using the Kaplan-Meier method, and statistical significance was determined by the log-rank test. A p-value of less than 0.05 was considered statistically significant.

**References:**

[1]	Duan Y, Zhan H, Wang Q, et al. Integrated Lactylome Characterization Reveals the Molecular Dynamics of Protein Regulation in Gastrointestinal Cancers [J]. Adv Sci (Weinh), 2024, 11(35): e2400227.

[2]	Cancer Genome Atlas Research N. Comprehensive molecular characterization of gastric adenocarcinoma [J]. Nature, 2014, 513(7517): 202-9.

[3]	Goldman M J, Craft B, Hastie M, et al. Visualizing and interpreting cancer genomics data via the Xena platform [J]. Nat Biotechnol, 2020, 38(6): 675–8.

[4]	Gyorffy B. Integrated analysis of public datasets for the discovery and validation of survival-associated genes in solid tumors [J]. Innovation (Camb), 2024, 5(3): 100625.

[5]	Uhlen M, Zhang C, Lee S, et al. A pathology atlas of the human cancer transcriptome [J]. Science, 2017, 357(6352).

[6]	Uhlen M, Fagerberg L, Hallstrom B M, et al. Proteomics. Tissue-based map of the human proteome [J]. Science, 2015, 347(6220): 1260419.

[7]	Wang D, Qian X, Du Y N, et al. cProSite: A web based interactive platform for online proteomics, phosphoproteomics, and genomics data analysis [J]. J Biotechnol Biomed, 2023, 6(4): 573-8.
